# Whole-genome and enzymatic analyses of an androstenedione-producing *Mycobacterium* strain with residual phytosterol-degrading pathways

**DOI:** 10.1186/s12934-020-01442-w

**Published:** 2020-10-02

**Authors:** Hongwei Wang, Shikui Song, Fei Peng, Fei Yang, Tian Chen, Xin Li, Xiyao Cheng, Yijun He, Yongqi Huang, Zhengding Su

**Affiliations:** 1 Key Laboratory of Industrial Fermentation (Ministry of Education), Hubei Key Laboratory of Industrial Microbiology, National “111” Center for Cellular Regulation and Molecular Pharmaceutics, Hubei University of Technology, Wuhan, 430068 China; 2 Wuhan Amersino Biodevelop Inc., B1-Building, Biolake Park, Wuhan, 430075 Hubei China; 3 Hubei Goto Biotech Inc., No. 1 Baiguoshu Road, Shuidu Industrial Park, Danjiangkou, 442700 Hubei China

**Keywords:** 1,4-Androstene-3,17-dione (ADD), 3-Hydroxy-9,10-secoandrost-1,3,5(10)-triene-9,17-dione (HSA), 3-Ketosteroid-1,2-dehydrogenase (KstD), 3-Ketosteroid-9α-hydroxylase (ksh), 4-androstene-3,17-dione (4-AD), 9-Hydroxyl-4-androstene-3,17-dione (9OH-AD), 21-Hydroxy-20-methylpregn-4-en-3-one (BA), Biotransformation, Cholesterol oxidases (cho), Monooxygenase (mon), *Mycobacterium* sp. strain

## Abstract

*Mycobacterium neoaurum* strains can transform phytosterols to 4-androstene-3,17-dione (4-AD), a key intermediate for the synthesis of advanced steroidal medicines. In this work, we presented the complete genome sequence of the *M. neoaurum* strain HGMS2, which transforms β-sitosterol to 4-AD. Through genome annotation, a phytosterol-degrading pathway in HGMS2 was predicted and further shown to form a 9,10-secosteroid intermediate by five groups of enzymes. These five groups of enzymes included three cholesterol oxidases (ChoM; group 1: ChoM1, ChoM2 and Hsd), two monooxygenases (Mon; group 2: Mon164 and Mon197), a set of enzymes for side-chain degradation (group 3), one 3-ketosteroid-1,2-dehydrogenase (KstD; group 4: KstD211) and three 3-ketosteroid-9a-hydroxylases (Ksh; group 5: KshA226, KshA395 and KshB122). A gene cluster encoding Mon164, KstD211, KshA226, KshB122 and fatty acid β-oxidoreductases constituted one integrated metabolic pathway, while genes encoding other key enzymes were sporadically distributed. All key enzymes except those from group 3 were prepared as recombinant proteins and their activities were evaluated, and the proteins exhibited distinct activities compared with enzymes identified from other bacterial species. Importantly, we found that the KstD211 and KshA395 enzymes in the HGMS2 strain retained weak activities and caused the occurrence of two major impurities, i.e., 1,4-androstene-3,17-dione (ADD) and 9-hydroxyl-4-androstene-3,17-dione (9OH-AD) during β-sitosterol fermentation. The concurrence of these two 4-AD analogs not only lowered 4-AD production yield but also hampered 4-AD purification. HGMS2 has the least number of genes encoding KstD and Ksh enzymes compared with current industrial strains. Therefore, HGMS2 could be a potent strain by which the 4-AD production yield could be enhanced by disabling the KstD211 and KshA395 enzymes. Our work also provides new insight into the engineering of the HGMS2 strain to produce ADD and 9OH-AD for industrial application.

## Introduction

Steroids, including phytosterols and cholesterol, are ubiquitous in nature and significant growth substrates for microorganisms. Steroids are biologically active organic compounds that function either as important components of cell membranes or as signaling molecules [1]. Typically, steroid medicinal compounds share a tetracyclic hydrocarbon ring or gonane structure with different functional groups attached to C17-site. Three androstane steroids, namely, 4-androstene-3,17-dione (4-AD), 1,4-androstadiene-3,17-dione (ADD) and 9α-hydroxy-4-androstenediol (9-OH-AD), are the most important starting materials that are used to synthesize advanced steroid medicines through chemical and/or enzymatic modification [2, 3]. Currently, one of the most attractive strategies producing these materials is to employ bacteria to transform phytosterols to androstane steroids [3, 4]. Aerobic side chain degradation of phytosterols is the basis for the production of androstane steroids. Bacteria belonging to the genera *Gordonia*, *Nocardia* and *Rhodococcus* are able to degrade the side chains of phytosterols [5–7]. To date, only strains in *Mycobacterium* genus have been found to have the potential to accumulate androstane steroids for industrial applications [2, 3, 8–10].

Many key enzymes in the steroid degradation pathways of pathogenic bacteria have been studied biochemically [11, 12], although the pathways are not fully understood. The importance of genomic analyses on steroid degradation pathways in microorganism has recently been drawn much attention [13]. Many genome sequences of pathogenic *Mycobacterium tuberculosis* strains are available [14], providing information guidance for understanding various steroid degradation pathways. However, genomic data on nonpathogenic mycobacteria, especially androstane steroid-producing strains, are still limited so far. *M. smegmatis* MC^2^ 155 is a nonpathogenic model to study drug resistance and shares significant genomic similarity with *M. tuberculosis* [15]. *Mycobacterium neoaurum* NRRL B-3805 was the first industrial strain used for the biotransformation of β-sitosterol to 4-AD [16]. Before its whole genome sequence became available [3, 17], biochemical analysis revealed that NRRL B-3805 transformed β-sitosterol to 4-AD via eleven catabolic enzymes acting in fourteen consecutive enzymatic steps to degrade the branched hydrocarbon side-chain of phytosterols [6, 18]. The global transcriptome analysis of *Mycobacterium* sp*.* VKM Ac-1817D, a strain producing 9OH-AD and genetically close to NRRL B-3805, revealed that phytosterols stimulated the increased expression of 260 genes, including those related to steroid catabolism in mycobacteria [19]. Other bacteria contain either a similar steroid degradation pathway, e.g., in *Pseudomonas* sp. *NCIB 10590* [20], or distinct steroid metabolic gene clusters, e.g., in *Nocardioides simplex* VKM Ac-2033D [4, 21]. Based on the Ref-Seq database, Mohn et al. recently identified 265 putative steroid-degrading bacteria that shared 9,10-secosteroid degradation as a converged pathway [13, 22]. This pathway was expanded to form cholesterol-, cholate- and testosterone-degrading pathways through gene duplication, horizontal gene transfer and plasmid facilitation. However, our understanding of the set of genes for specific androstane steroids that are involved in phytosterol biotransformation remains limited.

Genetic manipulation of the phytosterol-transformating strains has been attempted to improve transformation yield [2]. Knockout of the *kasB* gene for the cell wall synthesis of *M. neoaurum* ATCC 25795 strain enhanced the bioconversion of phytosterols to 9OH-AD by 1.4-fold [23]. Null mutation of 3-ketosteroid-9α-hydroxylase (Ksh) genes in the *M. neoaurum* strains led to the stable accumulation of androst-4-ene-3,17-dione (AD) and androst-1,4-diene-3,17-dione (ADD) [24–26]. Mutation in 4-AD-producing strain *M. phlei* M51 blocked 9α-hydroxylation, strain transformed β-sitosterol to 23,24-dinorcholane derivatives, which are useful starting materials for corticosteroid synthesis [27].

Among the aforementioned three important androstane steroids, 4-AD is one of the most important starting materials [28] in the synthesis of steroid medicines such as abiraterone [29], testosterone [30], estradiol [31] and progesterone [32]. Microbial bioconversion of phytosterols to 4-AD is still an industrial challenge. Although the complete steroid metabolic pathways of *Mycobacterium* strains are not fully understood, *Mycobacterium neoaurum* strains have been successfully utilized for the industrial production of 4-AD and 9OH-AD using β-sitosterol as substrate [2, 33, 34]. However, the transformation yields of currently used industrial strains remain unstable, as bioconversion fermentation is often accompanied by either complete degradation of the substrate or the generation of various metabolic impurities [3, 5]; therefore, further exploration of new 4-AD-producing strains is required.

In this work, we attempted to isolate and characterize phytosterol-degrading strains from soils contaminated with vegetable oils. One of the isolates, namely, *M. neoaurum* HGMS2, enabled the accumulation of 4-AD in vegetable oil-based media. Therefore, we were initially focused on elucidating the phytosterol degradation pathway of the strain by genome sequencing and enzymatic analysis. The key enzymes predicted to be involved in phytosterol degradation in HGMS2 were overexpressed using different *E. coli* protein expression systems to obtain soluble enzymes. The specific catalytic reaction of each key recombinant enzyme was evaluated by enzymatic activity assays, thin-layer chromatography (TLC) and high-performance liquid chromatography (HPLC). Finally, a concise outline of the phytosterol degradation pathway in *M. neoaurum* HGMS2 was generated. The results obtained from this work unveiled the causes of the low bioconversion rate and impurities in HGMS2 and other strains. Our work reveals the feasibility of engineering the HGMS2 strain in which the yield of 4-AD could be improved and the purification process could be enhanced by knocking out impurity-associated genes.

## Materials and methods

### Materials

A DNA gel extraction kit was purchased from Omega Biotek (Hubei, China). Other molecular biology reagents were of the highest grade and were obtained from Thermo Scientific (Shanghai, China). 4-Androstene-3,17-dione (4-AD), 1,4-androstene-3,17-dione (ADD) and 9-hydroxyl-4-androstene-3,17-dione (9OH-AD) with purities of 98% were obtained from Hubei Goto Pharmaceutical Co. (Xiangfan, China). 4-Cholesten-3-one, 4-cholesten-3-one-26-ol phenazine methosulfate (PMS, 99%) and nitro blue tetrazolium with purities of 99% were purchased from Sigma-Aldrich (Shanghai, China). Restriction enzymes, dNTPs, and *Taq* polymerase were purchased from TaKaRa Co. (Dalian, China). The Cho enzyme from *Streptomyces* sp. was prepared and purified by our laboratory.

### Isolation of microorganisms capable of degrading β-sitosterol

Samples were collected from soils contaminated with vegetable oils from Xiangyang suburb, Hubei, China. A soil sample of l.0 g was mixed with 1.0 g of autoclaved sand (50–70 mesh, Sigma-Aldrich, USA), added to 200 mL of autoclaved Milli-Q water and stirred for 30 min. The mixture was filtrated with autoclaved Whatman Grade 4 filter paper (Particle retention > 25 μm, GE, USA). The filtrate was mixed with 1% β-sitosterol and 10% skim milk powder and lyophilized overnight to reduce the sample volume. The lyophilized powder was resuspended in 20 mL of autoclaved Milli-Q water. The suspension was distributed in 0.2 mL aliquots, and each aliquot was spread on a MOPS agar plate containing 1% β-sitosterol as the sole carbon. All plates were incubated at 30 °C for 6 days, the growing microorganisms were isolated and amplified with LB medium. Isolated bacterial strains were maintained on LB agar slants for 3 days at 30 °C, and then stored at 4 °C and subcultured monthly. These strains were further screened for their genotypes with 16S ribosomal RNA (rRNA) sequence analysis, using two universal primers, i.e., 8F (5′-AGA GTT TGA TCC TGG CTC AG-3′) and 1492R (5′-CGG TTA CCT TGT TAC GAC TT-3′).

### Cell growth of *Mycobacterium* strains and extraction of fermentation metabolites

*Mycobacterium* strains were initially cultured in 5 mL of LB medium at 30 °C for 48 h and then used as seeds. When the OD_600nm_ value reached 15, the culture was inoculated into 50 mL of fermentation medium containing yeast extract (15 g/L), glucose (6 g/L), (NH_4_)_2_HPO_4_ (0.6 g/L), NaNO_3_ (5.4 g/L), β-sitosterol (80 g/L), Tween-80 (0.8 g/L), soybean oil (180 g/L) and lectin (3 g/L), and shaken at 30 °C and 200 rpm for 7 days. Then, 1 ml of fermentation broth was collected every 24 hs for the extraction of metabolites.

The fermentation broth was thoroughly mixed with ethyl acetate at a ratio of 1:1. The mixture was centrifuged at 8000×*g* for 5 min, and the supernatant was separated. The supernatant was collected and dried by heating using a hair drier. The dried sample was dissolved in an appropriate solvent for further assays.

### Genome analysis of the HGMS2 strain

Genomic DNA of the HGMS2 was extracted with genomic DNA preparation kits (Tiangen, China) and its quality was checked by agarose gel electrophoresis. Qualified DNA samples were sheared into smaller fragments with an average size of 500 bp by using a Covaris S/E210 device (Covaris, MA, USA). The overhangs resulting from fragmentation were converted into blunt ends by using T4 DNA polymerase, the Klenow fragment and T4 polynucleotide kinase. The blunt-ended fragments were ligated to adapters; and the desired fragments were purified by gel electrophoresis and selectively enriched and amplified by PCR. An index tag could be introduced into the adapter at the PCR stage if needed. The qualified bisulfite (BS) library was built for sequencing.

Genome sequencing of the HGMS2 strain was performed with the Illumina HiSeq 4000 platform (Illumina, CA, USA) and PacBio RSII platform (PACBIO, CA, USA) at BGI Genomics (Shenzhen, China) with standard procedures, as shown in Additional file 1: Figure S1a. Clean reads were obtained from the two platforms by filtering impure raw reads according to the protocols provided by the manufacturers (Additional file 1: Figures S1b, c). The continuous long reads were de novo assembled using Glimmer 2.0 [35]. Genomes were annotated using the NCBI Prokaryotic Genome Automated Pipeline, version 2.9, on the RAST annotation server. The metabolic pathways in the HGMS2 strain and the associated enzyme system were predicted through bioinformatics methods against the KEGG pathway database [36], GO database [37], COG database and Swiss-Prot database [38]. Animal pathogen analysis was performed against the Pathogen Host Interaction (PHI) database, Virulence Factors database (VFDB) and Antibiotic Resistance Gene Database (ARDB).

### Molecular cloning of predicted key genes

To obtain soluble protein, three kinds of fusion tags, namely, His6-tag, His6-GST-tag and His6-MBP-tag, were engineered into the pET28b(+) vector. The thrombin cleavage site was replaced with a TEV protease cleavage site. The three vectors were named pSZD, pGST and pMBP. To subclone each putative gene into the above pET-type protein expression vectors, a pair of restriction enzyme sites, namely, *BamH*I and *EcoR*I, was also introduced as a cloning site behind the DNA sequence encoding the fusion tag.

Four groups of key genes encoding cholesterol oxidase (Cho), 3β-hydroxyl-dehydrogenase (Hsd), 3-ketosterol-1,2-dehydrogenase (KstD), 3-ketosteroid-9α-hydroxylase (Ksh) and C26-monooxygenase (Mon) were amplified from the genomic DNA of HGMS2 using PCR. As the *Mycobacterium* strains had a high GC content, PCR was generally performed with a gradient temperature from 54 to 66 °C for annealing with an interval of 2 °C. For each PCR, a pair of primers including one forward primer and one reverse primer was used, as listed in Additional file 1: Table S1. Two restriction enzyme sites, i.e., *BamH*I and *EcoR*I, were introduced at the 5′- and 3′-ends of each PCR product, respectively. However, if the targeted genes contained these restriction enzyme sites, an alternative pair of restriction enzyme sites was used, as indicated in Additional file 1: Table S1. Each PCR product was purified using a gel purification kit and digested with BamHI and EcoRI, except those indicated in Additional file 1: Table S1. Each digested PCR product was ligated into the digested pSZD, pGST and pMBP vectors, and twenty-nine protein expression vectors were constructed (Additional file 1: Table S2).

Each plasmid was transformed into *E. coli* DH5α competent cells. The positive clones for each ligation reaction were picked and screened using colony PCR with the forward primer described above and the T7 terminator primer. The DNA sequence of each insert in a host vector was confirmed by DNA sequencing (BioSune, Shanghai, China).

### Protein expression and purification

Each plasmid was individually transformed into *E. coli* BL21(DE3) cells for protein expression. A single transformed colony was picked and inoculated into 1 mL of LB medium containing kanamycin (34 μg/mL). Cells were grown at 37 °C for 6 h and used as the seed culture. The seed culture was inoculated into 50 mL of fresh LB medium containing kanamycin (34 μg/mL) and incubated at 37 °C for 8 h. The 50-mL culture was then transferred into 1 L of fresh LB medium containing kanamycin (34 μg/mL). Cells were grown until the absorbance at 600 nm reached 0.8. The culture was cooled to 18 °C before 0.4 mM isopropyl-β-d-thiogalactopyranoside (IPTG) was added to induce protein expression. The cells were further grown at 18 °C with shaking at 200 rpm for 12 h. Before cells were harvested, small amounts of cell samples were collected and analyzed by SDS-PAGE to evaluate protein expression. To prepare SDS-PGAE samples, the OD_600nm_ of cell broth was adjusted to 1.0, and cells were pelleted at 5000×*g* for 10 min. Cell pellets were resuspended in in a buffer containing 50 mM K_2_HPO_4_, 200 mM NaCl, 10 mM imidazole and 1 mM β-mercaptoethanol (Buffer A, pH 8.0) and sonicated on ice using microtip with a pulse of 2 s burst and 10 s cooling until that cell suspension was just clarified. The lysed cell solution was centrifuged at 10,000×*g* for 10 min at 4 ℃. Forty microliters of supernatant was mixed with 10 μL of 5 × SDS-PAGE loading buffer (0.25 M Tris–HCl, pH 6.8, 10% SDS, 0.5% bromophenol blue, 50% glycerol, 5% β-mercaptoethanol). The cell pellet was resuspended with 50 μL of 1 × SDS-PAGE loading buffer. Both supernatant and pellet samples for SDS-PAGE were heated at 95 °C for 10 min and any precipitation was spun down.

Cells were harvested by centrifugation at 5000×*g* for 20 min, and the cell pellets were resuspended in Buffer A. The cell suspension was lysed by sonication and homogenization, followed by clarification using centrifugation at 18,000×*g* for 30 min. The supernatant was loaded onto a 10 mL Ni^2+^ affinity chromatography column (GE Healthcare, CT, USA), and the fusion protein was competitively eluted using a gradient of Buffer A mixed with Buffer B, which was the same as buffer A but contained 300 mM imidazole. The eluate containing the desired fusion protein was pooled and desalted with a prepacked GE desalting column, followed by either an SP-fast flow cation exchange column or Q-fast flow anion exchange column (GE Healthcare, CT, USA). An aliquot sample was taken from each fraction and subjected to SDS-PAGE analysis. SDS-PAGE was carried out using a 10–12% SDS-PAGE gel for 45 min at 200 V. The volume of each sample was 10 μL per well. The SDS-PAGE gels were stained with Coomassie Brilliant Blue staining solution with shaking for 30 min, followed by destaining with water overnight. Based on the SDS-PAGE results, the eluates containing enzymes were pooled for activity assays in this work without further purification.

Protein concentration was assayed by the Braford method with BSA as a standard [39]. The measurement was carried out with microplate-based procedures. Ten microliters of each standard or unknown sample was pipetted into the appropriate microplate wells, and 300 µL of Coomassie Plus Reagent was added to each well and mixed with a plate shaker for 30 s. The plate was incubated for 10 min at room temperature, and the absorbance was measured at 595 nm with a BioTek H1 plate reader (VT, USA). A standard curve was prepared by plotting the average blank-corrected measurement at 595 nm for each BSA standard vs. the corresponding concentration in µg/mL. The protein concentration of each unknown sample was determined by using the standard curve.

### Enzyme-catalyzed reaction and product extraction

Each enzyme-catalyzed reaction was performed in 100 mL Erlenmeyer flasks by mixing purified enzyme with homogenized substrate in 100 mM Tris–HCl buffer (pH 8.0) containing 10% glycerol. The final concentration of each substrate was 2 g/L. The reaction mixture was stirred with shaking at 200 rpm and 30 °C for 12–48 h. Samples were collected at 6 h intervals for extraction.

The products were extracted from the reaction solution by adding an equivalent volume of ethyl acetate and thoroughly shaken for 10 s. After phase separation, the upper organic phase was obtained as the sample extract and directly used for TLC and HPLC analyses.

### TLC analysis

TLC was performed on 0.25-mm-thick fluorescence-impregnated silica gel G (Silica gel 254, Haiyang Chemical Co., Qingdao, China) with a developing solvent system containing petroleum ether and ethyl acetate at a ratio of 6:4 (v/v) as the mobile phase. Sample extracts were spotted in 5-μL aliquots onto silica TLC plates. The products of the enzymatic reaction were visualized under ultraviolet (UV) light. If needed, the TLC plates were stained with 20% sulfuric acid at 100 °C for 10 min to identify compounds that were invisible under UV light.

### HPLC analysis

HPLC analysis was carried out on a C_18_ reverse-phase Sunfire column (5 μm, 4.6 × 150 mm, Waters, USA) at 30 °C with a Waters HPLC system equipped with a UV detector. Samples were diluted to an appropriate concentration (approximately 1 mg/mL) with methanol and filtered through 0.22-mm pore size membranes. HPLC was performed using a mixture of methanol and water at a ratio of 60:40 (v/v) as the mobile phase at a flow rate of 1 mL/min. Analytes were simultaneously detected with UV at 254 nm.

### Cholesterol oxidase activity assay

The activity of cholesterol oxidase (Cho) from the HGMS2 strain was spectrophotometrically determined by monitoring the generation of hydrogen peroxide during the cholesterol oxidation reaction according to the method of El-Naggar et al. [40]. In principle, to measure the enzyme activity, a horseradish peroxidase-coupled reaction was used to catalyze the conversion of hydrogen peroxide to oxygen to oxidize 4-aminoantipyrine and phenol and produce the dye quinoneimine, which exhibits maximum absorption at 500 nm. The reaction mixture contained 3 μM phytosterol in 1 mL of 1% Triton X-100, 0.1 mL of enzyme solution, 10 mM potassium phosphate buffer (pH 7.0), 1.2 μM 4-aminoantipyrine, 21 μM phenol and 20 U of horseradish peroxidase in a final volume of 3 mL. The reaction was initiated by adding enzyme solution into the reaction mixture. The enzyme reaction was performed at 37 °C for 30 min with shaking during the incubation period and terminated by boiling for 3 min. One unit of enzymatic activity (U) was defined as the amount of enzyme required to form one micromole of H_2_O_2_ per minute at 37 °C.

### Monooxygenase activity assay

Monooxygenase (Mon) activity was evaluated by monitoring substrate and product concentrations using HPLC analysis, according to the procedure described by Capyk et al. [41]. Reactions were conducted in multiple tubes containing 200 μL of the standard assay mixture. The mixture was prepared in 100 mM air-saturated potassium phosphate at pH 7.0 containing 300 μM NADH, 50 μM 4-cholesten-3-one, 1.5 μM KshB122, and 0.5 μM Mon164 or Mon174. Stock solutions of 1 mM 4-cholesten-3-one were made in 10% 2-hydroxypropyl-β-cyclodextrin (BCD), and stock solutions of 180 mM NADH were made fresh daily. Reactions were initiated by adding NADPH, and at each time point, the reaction was quenched by the addition of 200 μL of methanol, vigorous mixed and subjected to HPLC analysis.

Under these conditions, the elution times for 4-cholesten-3-one and 4-cholesten-3-one-26-ol were 30.5 and 26.4 min, respectively. The concentrations of 4-cholesten-3-one and 4-cholesten-3-one-26-ol were calculated from their respective peak areas using the standard curve of 4-cholesten-3-one and assuming that the extinction coefficients for the two compounds were similar. The *R*^2^ value for the standard curve was 0.99.

### 3-Ketosteroid-1,2-dehydrogenase activity assay

The activity of 3-ketosteroid-1,2-dehydrogenase (KstD) was measured spectrophotometrically at 600 nm for 10 min at 30 °C using PMS and DCPIP as artificial electron acceptors. The reaction was conducted in 500 μL of mixture solution containing 50 mM Tris–HCl buffer (pH 7.0), 1.5 mM PMS, 40 μM DCPIP and 250 mM AD in 2% methanol. The reaction was initiated by adding an appropriate concentration of partially purified KstD211. A reaction lacking AD was used as a control. Activity was expressed as U/mg of protein after subtracting control data. One unit (U) of enzyme activity was defined as the amount of enzyme required to reduce 1 μmol of DCPIP per minute (ε _600 nm_ = 18.7 × 10^3^ L/mol cm). The relationship between enzyme activity and pH was measured using citric acid-phosphate buffers (pH 5 to 7) and Tris–HCl buffers (pH 7 to 9).

### 3-Ketosteroid-9a-hydroxylase activity assay

The assay of 3-ketosteroid-9a-hydroxylase (Ksh) activity was performed by as previously described by Petrusma et al. with modification [42]. Briefly, the reaction mixture contained 500 μL of air-saturated 50 mM Tris–HCl buffer (pH 7.0), 100 μM NADH and 20 μg of enzyme. The reaction was initiated by the addition of 250 μM 4-AD into the mixture. The substrate solution was prepared by dissolving 4-AD powder in methanol. All assays were performed at 25 °C. A kinetic program on a NanoDrop C2000 spectrophotometer was used to continuously record NADH oxidation at 340 nm (ε = 6.22 L/mol cm).

The conversion of 4-AD to 9OH-AD was also evaluated by monitoring substrate and product concentrations using an HPLC system equipped with a Waters 2945 UV detector and a 150 × 4.6 mm C18 reverse-phase 5 μm ODS-analytical column (*see above*). In this work, the elution times for 4-AD and 9OH-AD were 10.6 and 4.4 min, respectively. The concentrations of 4-AD and 9OH-AD were calculated from their respective peak areas using standard curves of 4-AD and 9OH-AD, respectively. The *R*^2^ value for the standard curve was 0.99.

## Results

### Identification of *Mycobacterium* strains

To search for new microorganisms that were able to transform phytosterols into 4-AD, we mainly focused on the isolation of *Mycobacterium* strains from the soils that contaminated with vegetable oil residues for the following reasons. One reason was that vegetable oil residues are rich in phytosterols. Another was that *Mycobacterium* strains containing mycolic acids could survive in oil-based fermentation media. Moreover, the vegetable oil-based biotransformation technology of phytosterols has been well established at the industrial level.

Using minimum media containing 1% β-sitosterol as the sole carbon and energy source (see “Materials and methods”), we were able to isolate 12 bacterial strains from the soils. The genotypes the strains were identified by 16S ribosomal RNA (rRNA) sequence analysis (see “Materials and methods”). At this stage, only the *Mycobacterium neoaurum* strain was kept for further investigation. Any isolate that was possibly pathogenic was removed, as the most important pathogenic mycobacteria, such as the *M. tuberculosis* complex, *M. leprae*, and *M. avium* have different *rrn* operons [43]. Thus, four kinds of *M. neoaurum* strains were finally identified, including *M. neoaurum* HGMS1, HGMS2, HGMS3 and HMGS4.

**Fig. 1:**
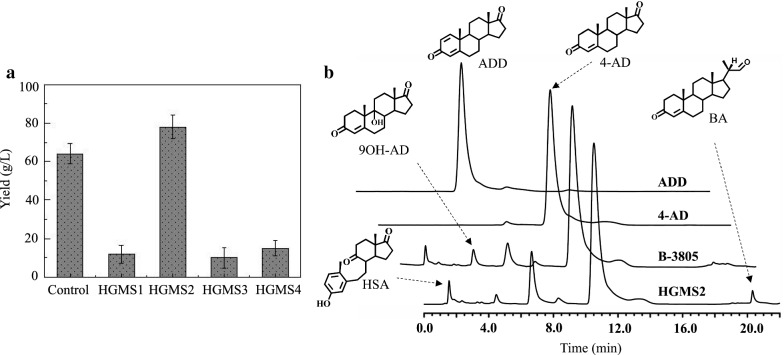
Isolation and screening of β-sitosterol-degrading strains. **a** Conversion rates of four HGMS strains for β-sitosterol were compared with that of the *M. neoaurum* NRRL B-3805 strain. **b** HPLC analysis of extracts from the fermentation broth. HAS, 3-hydroxy-9,10-secoandrost-1,3,5(10)-triene-9,17-dione; 9OH-AD, 9-hydroxyl-4-androstene-3,17-dione; ADD, 1,4-androstene-3,17-dione; 4-AD, 4-androstene-3,17-dione; BA, 1-hydroxy-20-methylpregn-4-en-3-one. These peaks were identified by mass spectrometry

The four HGMS strains were directly examined for their ability to transform β-sitosterol to 4-AD with fermentation media (see “Materials and methods”). Metabolites were extracted with solvent and evaluated with HPLC. Figure 1a summarizes the transformation yields of four HGMS2 strains, compared with B-3805. Notably, of the four HGMS2 strains, HGMS2 exhibited the highest yield (78 ± 4.3 g/L), and its yield was higher than that of B-3805.

HPLC profiles of the metabolites from HGMS2 were compared with those of the B-3805 strain and standard 4-AD and ADD samples (Fig. 1b). As indicated in Fig. 1b, HGMS strain could transform β-sitosterol to 4-AD as efficiently as B-3805. Both HGMS2 and B-3805 also produced other downstream intermediates of phytosterol degradation pathway, including ADD, 9OH-AD, HSA and BA. These intermediates accumulated during the fermentation process and reduced the 4-AD production yield [44]. However, it was notable that HGMS2 produced more ADD and less 9OH-AD than B-3805. One obvious difference was that the HGMS2 strain produced more BA than B-3805 strain. The identities of the HPLC peaks corresponding to 4-AD, ADD, 9OH-AD, BA and HSA were identified by MS assays (Additional file 1: Figure S2). These observations showed that both HGMS2 and B-3805 strains contain active enzymes that convert 4-AD to ADD and 9OH-AD. It was also likely that BA accumulated during side-chain degradation through a possibly different branched pathway. Therefore, it was worthwhile to characterize the genomic properties of phytosterol catabolic pathways in the HGMS2 strain and to identify its key enzymes that caused some impurities.

### Comparative genomics of *Mycobacterium* sp. HGMS2

**Fig. 2:**
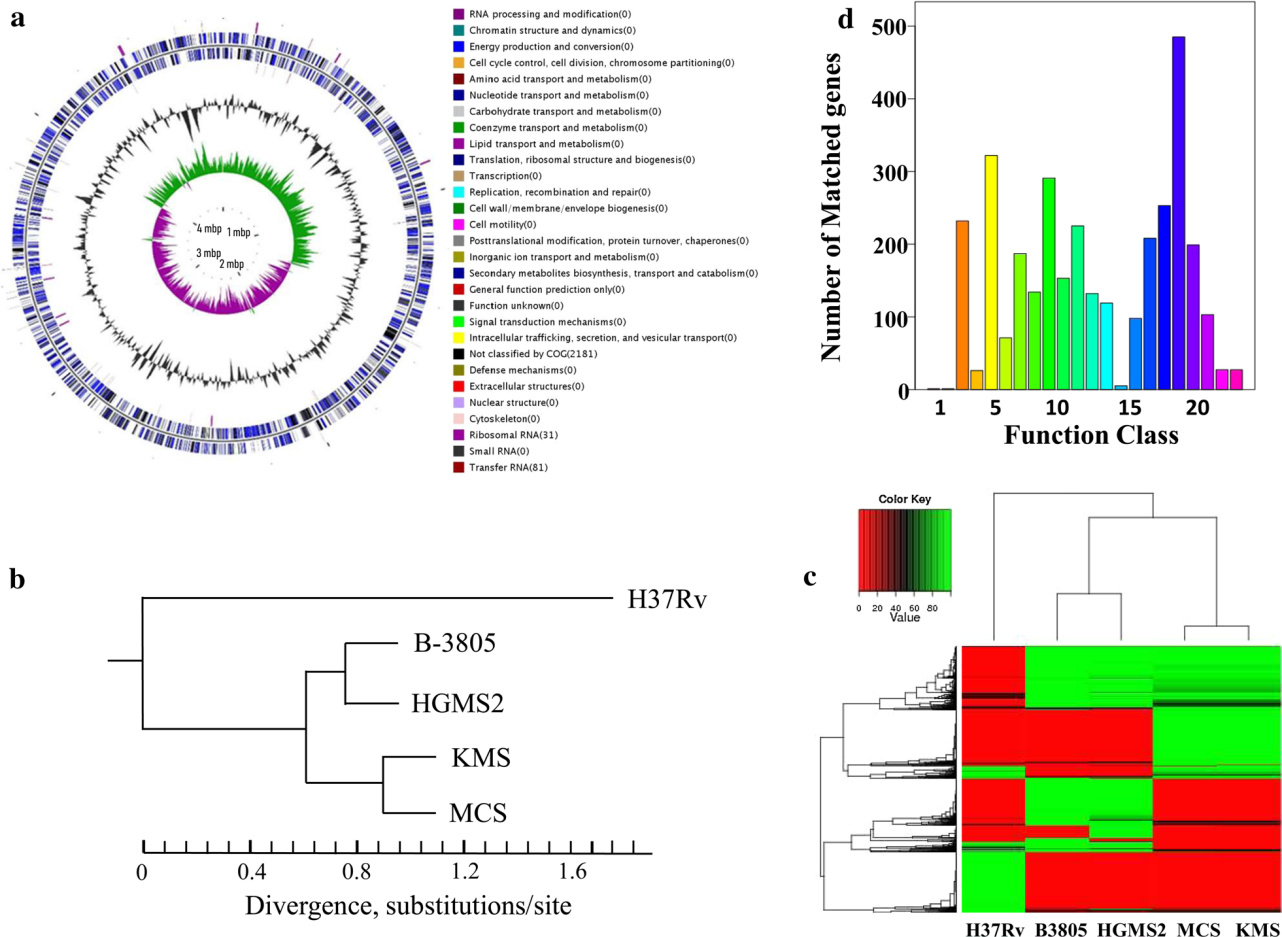
Genomic analysis of *Mycobacterium* sp. HGMS2. **a** Distribution of annotated ncRNA genes, COG gene, GC content and GC skew of *M. neoaurum* HGMS2. From outside to inside, the first circle indicates the distribution of ncRNA in the positive strand, including tRNA, three types of rRNA and sRNA (refer to the color codes on the right side); the second circle represents the distribution of the positive-stranded COG annotation genes, distinguished by different colors; the third circle represents the negative strand, the distribution of the COG annotated genes; the fourth circle indicates the distribution of the negative-stranded ncRNA; the fifth circle (black) indicates the GC content, with the average GC as the baseline, where the outwardly protruding means higher than the average, the inwardly protruding means lower than the average; the sixth circle is the GC skew value, where purple means less than 0, green means more than 0. **b** Heatmap comparison of the special genes of HGMS2 with those from four other mycobacteria, i.e., *Mycobacterium* sp. H37Rv, *Mycobacterium* sp. NRRL B-3805 (as reference), *Mycobacterium* sp. MCS and *Mycobacterium* sp. KMS. **c** Phylogenetic tree comparing HGMS2 with the aforementioned four mycobacteria. **d** Distribution of functional genes in the HGMS2 genome predicted by KEGG. A detailed classification of genes is summarized in Additional file 1: Table S4

We used the Illumina HiSeq 4000 and PacBio sequencing platforms to annotate the genomic sequence of HGMS2. The data showed that the single base analysis quality level was 0.9998, the structural base analysis quality was 0.9920, the readability was 0.9842, and the repetition rate was 1.35%. The general features are summarized in Table 1. The HGMS2 genome contained 5,421,383 bp and 6921 genes (Fig. 2a). Among these genes, there were 5139 protein coding genes. The total length of the protein coding genes was 4,993,092 bp, constituting 92.10% of the genome. The number of tandem repeat sequences was 420, and the total length of the tandem repeat sequences was 21,922 bp, constituting 0.4044% of the genome. The numbers of minisatellite DNAs, microsatellite DNAs, tRNAs and rRNA were 330, 20, 46 and 6, respectively.

**Table 1:** Summary of complete genome of *Mycobacterium neoaurum* HGMS2

Genome size (bp)	5,421,383
GC content (%)	66.88
Coding region (%)	92.10
Gene number	6921
Gene length (bp)	4,993,092
Protein coding genes	5139
Average CDS length (bp)	972
rRNA	6
5S	2
16S	2
23S	2
sRNA	3
tRNA	46
ncRNA	1
Tandem repeats	420
Minisatellite DNA	330
Microsatellite DNA	20
GenBank accession no	CP031414.1

Using the *M. neoaurum* B-3805 strain as a reference, we found that the HGMS2 genome exhibited over 88.1% sequence identity to the reference sequence (Additional file 2: Table S3). The genome of the HGMS2 strain contained 37,108 SNPs including 28,324 synonymous mutations and 1108 nonsynonymous mutations. However, we found that *M. tuberculosis* H37Rv contained 26,812 SNPs, including 17,388 synonymous mutations and 8912 nonsynonymous mutations. *M.* sp. KMS contained 20,284 SNPs including 13,525 synonymous mutations and 6344 nonsynonymous mutations. *M.* sp*.* MCS contained 20,284 SNPs including 13,525 synonymous mutations and 6344 nonsynonymous mutations. A phylogenetic tree for these queried strains was constructed (Fig. 2b). The genome sequencing results indicated that HGMS2 was an *M. neoaurum* strain. The genome features of HGMS2 were close to those of reference strain NRRL B-3805 and the strain MCS.

Protein functions and sequences encoded by the HGMS2 genes were annotated based on the Orthologous Groups (COG) database, and their positions in metabolic pathways were annotated based on the KEGG database. The functional genes of HGMS2 were classified into twenty-four groups (Fig. 2c) and are summarized in Additional file 1: Table S4. The detailed metabolic pathways of HGMS2 were mapped by comparison with the databases within and outside of *Mycobacterium* group and are summarized in Additional file 3: Figure S3. The genome of HGMS2 contained 6921 pangenes, including 3327 core genes (Additional file 1: Table S5). When the HGMS2 genome was compared with the genomes of four typical mycobacteria, i.e., *M.* B-3805, *M.* KMS, *M.* MCS and *M.* H37Rv, it was found that these mycobacteria shared significant core genes that were necessary for growth. Special genes in HGMS2 were classified by removing core genes from its pangenes and used to construct a heatmap (Fig. 2d). The heatmap showed the similarities and differences among the five mycobacterial genomes and confirmed that the genotype of the HGMS2 strain was closer to the B-3805 strain than the others. Importantly, we also analyzed the HGMS strain against the Pathogen Host Interaction (PHI) database, Virulence Factors database (VFDB) and Antibiotic Resistance Genes database (ARDB). The results are listed in Additional file 4: Table S6, Additional file 5: Table S7, Additional file 6: Table S8, indicating that HGMS2 was nonvirulent.

### Prediction of the phytosterol catabolic pathway in *Mycobacterium* sp. HGMS2

**Fig. 3:**
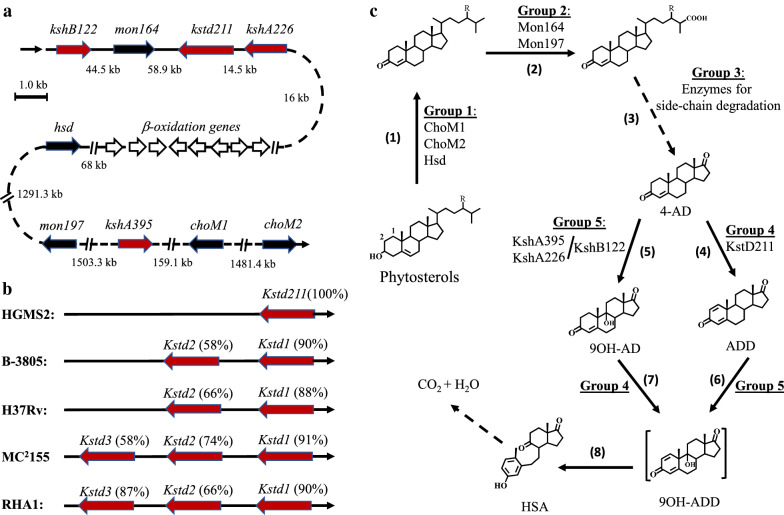
Predicted phytosterol-degrading pathway in *Mycobacterium* HGMS2. **a** Gene cluster of the key enzymes in the predicted phytosterol-degrading pathway. Black solid arrows, groups 1 and 2 genes; open arrows, group 3 genes and red solid arrows: groups 4 and 5 genes. The gaps of genes are marked as base pairs. **b** Numbers of KstD genes in different mycobacteria and the percentages of their nucleotide identity compared with KstD211. **c** A predicted phytosterol pathway was involved in five groups of key enzymes

In particular, the sterol catabolic gene clusters in *M. neoaurum HGMS2* were identified, as schematically described in Fig. 3a. These genes were annotated as orthologs of the genes from *M. tuberculosis* H37Rv. Essentially, these genes encoded two cholesterol oxidases (ChoM) and one 3β-hydroxyl-dehydrogenase (Hsd), two monooxygenases (Mon), a group of eleven fatty acid β-oxidoreductases, one 3-ketosteroid-1,2-dehydrogenase (KstD) and three 3-ketosteroid-9α-hydroxylases (Ksh). Compared with other previously studied strains, one of significantly different features of HGMS2 was that this strain contained one KstD gene (Fig. 3b). A putative phytosterol metabolic pathway in the HGMS2 strain was predicted (Fig. 3c). As shown in Fig. 3c, HGMS2 could transform phytosterols to 4-AD through the activity of the first 3 groups of enzymes or the first three kinds of reaction steps. The first reaction involved oxidation of the 3-hydroxy group to a carbonyl group by group 1 enzymes, while the 5,6-position double bond was allosterically shifted to the 4,5-position. Group 1 enzymes included two cholesterol oxidases (ChoM1 and ChoM2) and one 3β-hydroxyl-dehydrogenase (Hsd). It is well known that the oxidation of the 3-hydroxyl group is required for phytosterol to enter cells for further degradation. The second reaction was the initiation of side-chain degradation by group 2 enzymes, which included two monooxygenases (Mon), to catalyze the formation of a C26-hydroxyl group. Group 3 enzymes included a series of fatty acid β-oxidoreductases, which generally occurred in multiple reaction steps.

As shown in Fig. 3c, the HGMS2 strain was able to degrade phytosterols completely, and 4-AD should be just an intermediate in the pathway. Notably, our genomic sequencing analysis revealed the existence of 3-ketosteroid-1,2-dehydrogenase (KstD, group 4) and 3-ketosteroid-9α-hydroxylase (Ksh, group 5) in the HGMS2 strain. If these two groups of enzymes were active, the HGMS2 strain could degrade 4-AD by forming a transient intermediate, 9OH-ADD (Steps 6 and 7 in Fig. 3c). The resulting intermediate was extremely unstable and underwent simultaneous cleavage of the B-ring accompanied by aromatization of the A-ring to generate a secosteroid, i.e., 3-hydroxy-9,10-seco-androsta-1,3,5(10)-triene-9,17-dione (HSA, Step 8). HSA is readily metabolized to CO_2_ and H_2_O [11, 45]. Therefore, in the next step, we focused on evaluating the activities of these key enzymes predicted based on genomic sequencing data, except for the enzymes in group 3.

### Enzymatic characterization of cholesterol oxidases in group 1

**Fig. 4:**
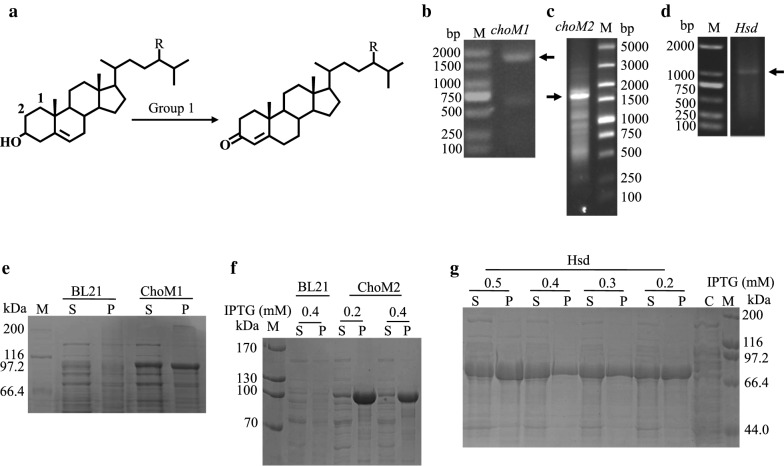
Preparation of soluble cholesterol oxidases. **a** Group 1 enzymes catalyze an oxidization reaction for the conversion of phytosterols to 4-cholesten-3-one. **b**–**d** Genes encoding ChoM1, ChoM2 and Hsd were amplified from the HGMS2 genome by PCR. Arrows indicate expected PCR bands. **e**–**g** The ChoM1 (**e**), ChoM2 (**f**) and Hsd (**g**) enzymes were overexpressed in soluble form and fused with His6-MBP. S, supernatant; P, pellet; C, total protein of *E. coli* BL21(DE3) cells as a control; M, molecular weight marker

HMGS2 contained three putative enzymes responsible for oxidizing the 3-hydroxyl group of phytosterols, namely, ChoM1, ChoM2 and Hsd in group 1 (Fig. 4a). As shown in Fig. 4b, the expected gene sizes of ChoM1 (1746 bp), ChoM2 (1617 bp) and Hsd (1101 bp) were amplified by PCR using the HGMS2 genome as a template. These genes were subcloned into modified protein expression vectors to obtain soluble enzymes (see “Materials and methods”). Three different fusion tags, from a small tag to a large tag, i.e., His6-tag, His6-GST-tag and His6-MBP-tag, were examined until a relatively high yield of soluble fusion protein was achieved.

**Fig. 5:**
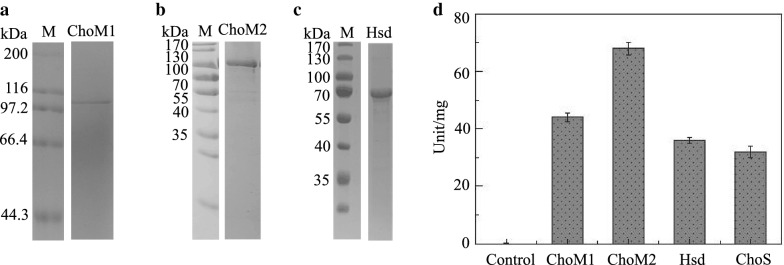
Comparison of the enzyme activities of ChoM1, ChoM2 and Hsd with that of ChoS. **a**–**c** SDS-PAGE assay of purified ChoM1, ChoM2 and Hsd. All molecular weight markers were run on the same gel. **d** The enzyme activities of ChoM1, ChoM2 and Hsd were compared with that of ChoS. *E. coli* BL21 (DE3) cell lysate was used as a negative control. Error bars indicate standard errors of the means of three repeat experiments

We found that the ChoM1, ChoM2 and Hsd enzymes could be expressed in soluble form in reasonable amounts when tagged with only the His6-MBP-tag (Fig. 4e–g), while their fusion proteins tagged with His-tag or His6-GST-tag formed in inclusion bodies (Additional file 1: Table S2). Three fusion proteins tagged with His6-MBP-tag were partially purified with a Ni–NTA affinity chromatographic column (Fig. 5a–c). The purified His6-MBP-ChoM1, His6-MBP-ChoM2 and His-MBP-Hsd enzymes were used for activity assays using β-sitosterol as the substrate and MBP as a negative control. Their activities were monitored at 500 nm to determine the production rate of hydrogen peroxide via a coupled enzyme reaction. As shown in Fig. 5d, the three enzymes exhibited significant β-sitosterol oxidization activity (Table 2), compared with the positive control, Cho enzyme (ChoS) from *Streptomyces* sp. [46]. Among our three Cho enzymes, ChoM2 had the highest activity.

**Table 2:** Specific activities of key enzymes in comparison with others

Key enzymes	Specific activity (unit/mg)	Data from references	
ChoM1	42.6 ± 2.1	32 ± 2.6 31 ± 1.7	ChoS [40] ChoS (this work)
ChoM2	68.4 ± 3.6		
Hsd	31.4 ± 2.2		
MO164	68.5 ± 5.2	23.5 ± 4.6	Cyp125 [41]
MO197	47.2 ± 7.4		
KstD211	125.7 ± 23.4	8883.0 ± 791	KstD2 [53]
KshA226	ND (4-AD)	261 ± 11 (4-AD)	KshA1 [54]
KshA395	56.4 ± 7.9 (4-AD)		
KshB122	ND (4-AD)		

ND: no activity was detectable

### Enzymatic characterization of monooxygenase in group 2

**Fig. 6:**
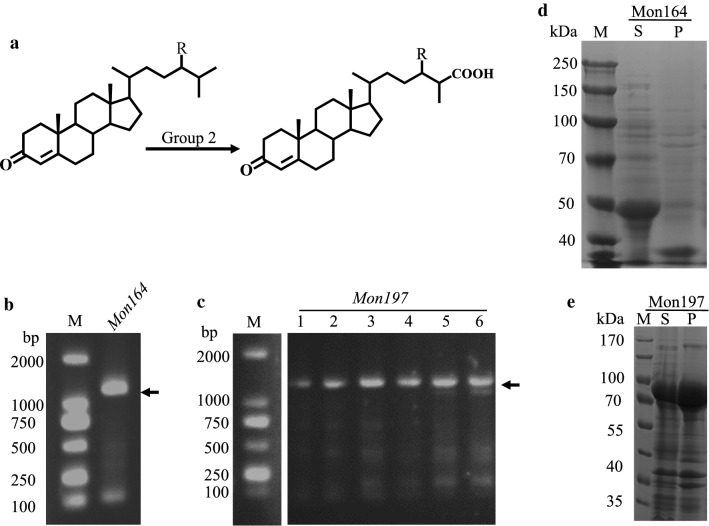
Preparation of soluble monooxygenases. **a** Group 2 enzymes catalyze an oxidization reaction of phytosterol at the C26 position. **b** PCR amplification of the Mon164 gene from the HGMS2 genome indicated the expected sizes. The arrow indicates the expected PCR band. **c** PCR amplification of the Mon197 gene from the HGMS2 genome with a gradient annealing temperature. The arrow indicates the expected PCR band. The DNA marker was run on the same gel in **c. d** Mon164 was overexpressed in soluble form and fused with a His-tag. S, supernatant; P, pellets and M, molecular weight marker. **e** Mon197 was overexpressed in soluble form in fusion with a His6-MBP-tag. S, supernatant; P, pellet and M, molecular weight marker

Monooxygenase (Mon) from mycobacteria is a terminal oxidase of the cytochrome p450 enzyme family. Mon contains an iron porphyrin prosthetic group and catalyzes the regio- and stereo-specific oxidation of nonactivated hydrocarbons under mild conditions. As shown in Fig. 6a, Mon can catalyze the oxidation of the C-terminus of phytosterols to form a hydroxyl group. In the HGMS2 strain, two putative Mons were predicted and named Mon164 and Mon197. The genes encoding Mon164 and Mon197 were amplified from the HGMS2 genome (Fig. 6b, c). The amplified genes were subcloned into our fusion protein expression vectors for evaluation of their activities. As shown in Fig. 6c, d, Mon164 was expressed as a soluble protein when tagged with His6-tag, while Mon197 was expressed in soluble form only when tagged with His6-MBP-tag, i.e., His6-MBP-Mon197.

**Fig. 7:**
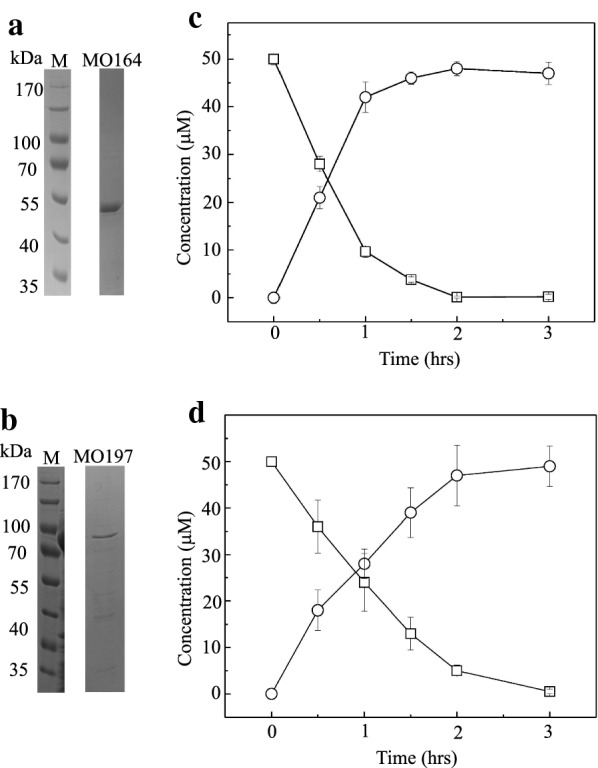
Assay of the substrate conversion catalyzed by Mon164 and Mon197. **a**, **b** SDS-PAGE assay of purified Mon164 and Mon197. M, molecular weight marker. All molecular weight markers were run on the same gel. **c**, **d** Turnover of 4-cholesten-3-one by Mon164 and Mon197, respectively, as analyzed by HPLC. *Squares*, 4-cholesten-3-one; *circles*, 26-hydro-4-cholesten-3-one. Error bars indicate standard errors of the means of three repeat experiments

Two fusion proteins, i.e., His6-Mon164 and His6-MBP-Mon197, were partially purified with a Ni–NTA affinity chromatographic column (Fig. 7a, b). Purified His6-Mon164 and His6-MBP-Mon197 enzymes were used for activity assays by HPLC using 4-cholesten-3-one as the substrate and 4-cholesten-3-one-26-ol as the product reference. As shown in Fig. 7c, d, these two enzymes exhibited significant activities for the oxidization of 4-cholesten-3-one to 4-cholesten-3-one-26-ol, compared with Cyp125 (Table 1). Notably, His6-Mon164 was more active than His-MBP-Mon197 and was able to oxidize 4-cholesten-3-one to 4-cholesten-3-one-26-ol within 2 h.

### Enzymatic characterization of 3-ketosteroid-1,2- dehydrogenase in group 4

**Fig. 8:**
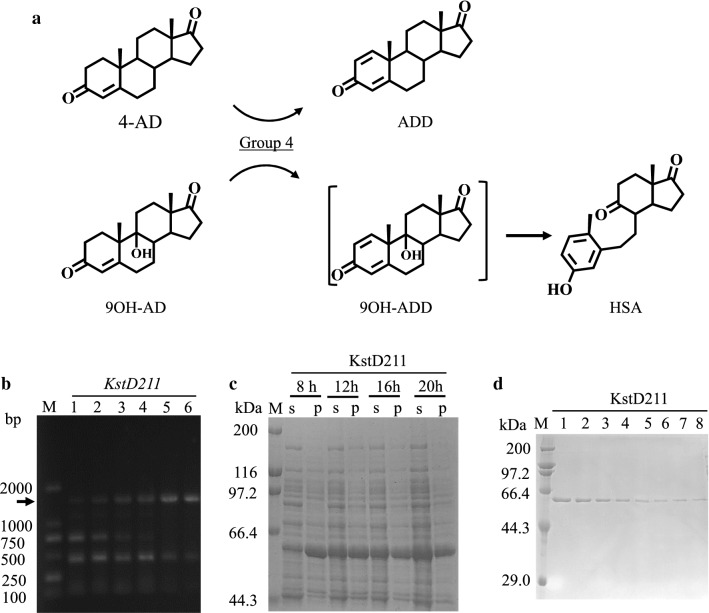
Preparation of soluble KstD211. **a** Group 4 contains one enzyme catalyzing dehydrogenation at the C1,2-position of 4-AD to generate ADD. **b** The KstD211 gene was amplified from the HGMS2 genome by PCR with a gradient annealing temperature. Arrows indicate expected PCR bands. **c** KstD211 was overexpressed in soluble form with a His6-tag at 18 °C. S, supernatant; P, pellets and M, molecular weight marker. **d** SDS-PAGE visualization of KstD211 purified by Ni–NTA affinity chromatography followed by MonoS chromatography. Lanes 1–8 represent samples from different fractions

KstD is a key enzyme required for phytosterol degradation by C1,2-position dehydrogenation. KstD can utilize either 4AD or 9OH-AD as a substrate (Fig. 8a). In the HGMS2 genome, we were able to annotate only one putative KstD gene, namely, KstD211 in group 4, although multiple KstD genes were identified in other *Mycobacterium* strains (Fig. 3b). The deduced amino acid sequence of KstD211 was distinct from those of previously reported acetobacterium KstDs (Additional file 1: Figure S4). Nevertheless, KstD211 was similar to KstDs within the mycobacterium genus, including the B-3805, KMS, H37Rv and MCS strains (Additional file 1: Figure S5). Thus, based on sequence homology, KstD211 should have certain activity, as many key residues in its active site are conserved.

**Fig. 9:**
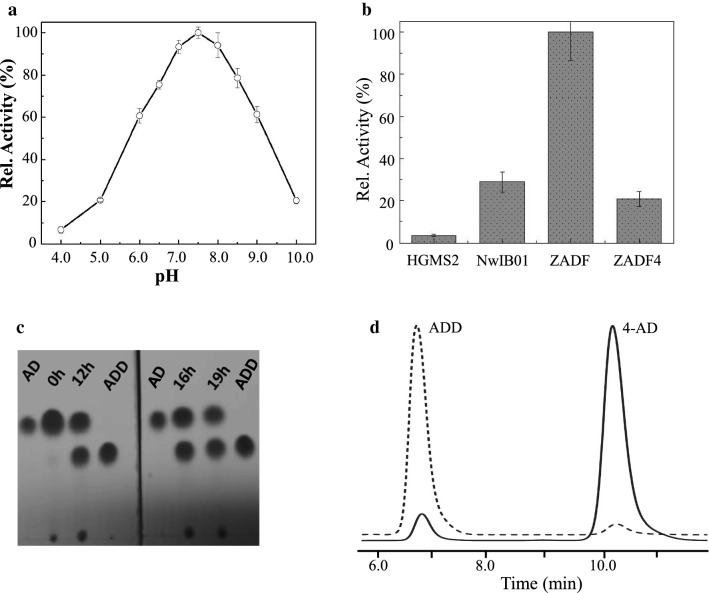
Assay of the substrate conversion catalyzed by KstD211. **a** Enzyme activity of KstD211 as a function of pH value. **b** Comparison of the enzyme activity of KstD211 with that of its homologues. NwIB01, ZADF and ZADF4 represent KstD enzymes from *Mycobacterium* NwIB01, *Mycobacterium* ZADF and *Mycobacterium* ZADF4, respectively. **c** TLC assay of the conversion of 4-AD to ADD catalyzed by KstD211 within 19 h. **d** HPLC analysis of KstD211-catalyzed turnover of 4-AD to ADD after 48 h. Solid and dashed lines represent HPLC profiles of reaction mixtures at the beginning and the end of reaction, respectively, for 48 h. The conversion rate was calculated based on regression using the area under the curves for two peaks. Error bars indicate standard errors of the means of three repeat experiments

The KstD211 gene was amplified by PCR using the HGMS2 genome as a template (Fig. 8b). The amplified gene was subcloned into the *E. coli* protein expression system using three types of fusion tags: His-tag, His6-GST-tag and His6-MBP-tag. The KstD211 fusion protein was overexpressed as a His-tagged fusion protein (Fig. 8c). After removing the His-tag by TEV protease cleavage, KstD211 was purified by an ion exchange chromatographic column (Fig. 8d). Purified enzymes exhibited optimal activity at approximately pH 7.5 using 4-AD as the substrate (Fig. 9a). However, the activity was much weaker than those of KstDs from other bacteria (Fig. 9b and Table 1). Nevertheless, KstD211 converted 50% of the 4-AD to ADD within 19 h, as determined by the TLC assay (Fig. 9c). As shown in Fig. 9d, this kind of activity could convert 97% of 4-ADD to ADD within 48 h, as evaluated by the HPLC assay.

### Enzymatic characterization of 3-ketosteroid-9α-hydroxylase in group 5

**Fig. 10:**
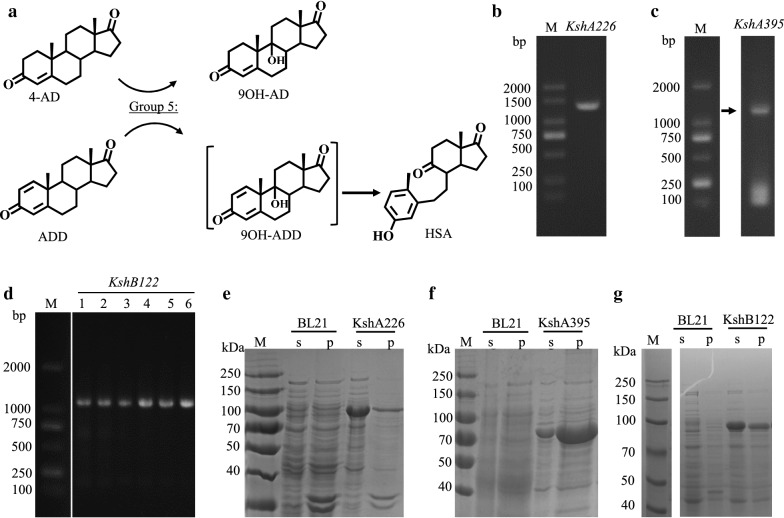
Preparation of soluble KshA226, KshA395 and KshB122. **a** Group 5 enzymes can use either 4AD or ADD as substrates to form 9OH-AD or 9OH-ADD, respectively, while 9OH-ADD can simultaneously be converted to HSA through a 9,10-secoreaction. **b**, **c**. Agarose gel images of products of the KshA226 and KshA395 genes amplified from the HGMS2 genome by PCR, respectively, indicating the expected sizes. M, molecular weight marker; arrows indicate expected PCR products. **d** Agarose gel image of the KshB122 gene amplified from the HGMS2 genome by PCR, indicating the expected size. M, DNA marker, which was run on the same gel; lanes 1–6, different PCRs with a gradient of annealing temperatures from 54 to 66 °C with an interval of 2 °C. **e**–**g** Overexpression of KshA226, KshA395 and KshB122 was evaluated by SDS-PAGE

**Fig. 11:**
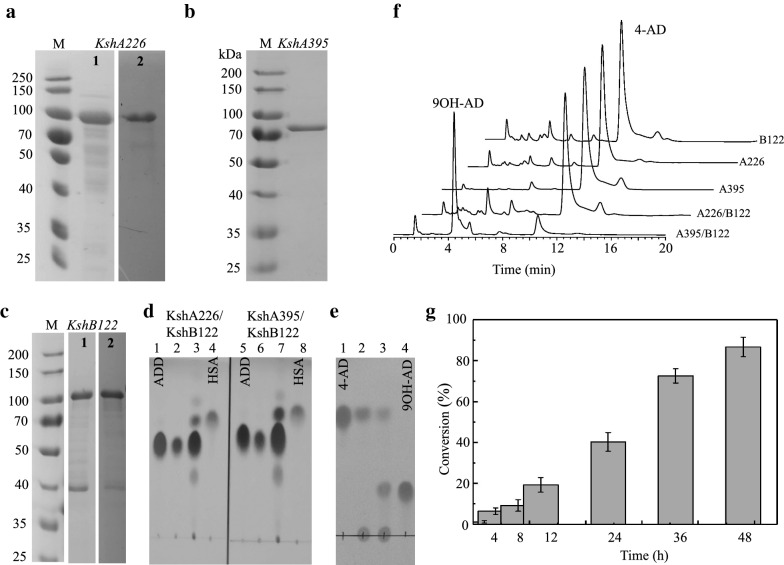
Assay of the substrate conversion catalyzed by KshA226 and KshA395 in combination with KshB122. **a**–**c** SDS-PAGE evaluation of purified KshA226, KshA395 and KshB122 fused with MBP. M, Molecular weight markers, which were run on the same gel. In **a**, sample 2 was run on a different gel simultaneously with the same electrophoresis conditions. In **c**, sample 2 was run on a different gel simultaneously. **d** TLC assay of the conversion of ADD to HSA catalyzed by KshA226 and KshA395, respectively, in combination with KshB122. Lanes 1 and 5, ADD standard; lanes 4 and 8, HSA standard; lanes 2 and 6, the reaction mixtures catalyzed by KshA226/KshB122 and KshA395/KshB122, respectively, for 5 h; and lanes 3 and 7, the reaction mixtures catalyzed by KshA226/KshB122 and KshA395/KshB122, respectively, for 20 h. **e** TLC evaluation of the conversion of 4-AD to 9OH-AD catalyzed by KshA226 or KshA395 in combination with KshB122 within 20 h. Lane 1, 4-AD standard; lane 4, 9OH-AD standard; lanes 2 and 3, KshA226/KshB122 and KshA395/KshB122, respectively, for 5 h; **f** HPLC assay of the conversion of 4-AD to 9OH-AD catalyzed by KshA226 or KshA395 in combination with KshB122 for 40 h. The peaks for 4-AD and 9OH-AD were marked. **g** Enzyme-catalyzed conversion of 4-AD to 9OH-AD by KshA395 in combination with KshB122 for 40 h. Error bars indicate standard errors of the means of three repeat experiments

We were able to identify three Ksh genes in the HGMS2 genome, namely, KshA226, KshA395 and KshB122. KshA226 and KshA395 were expected to catalyze 3-ketosteroid-9α-hydroxylation in combination with KshB122 (Fig. 10). To characterize their enzymatic properties, the Ksh genes were amplified from the HGMS2 genome and subcloned into our fusion protein expression vectors (see “Materials and methods”). These enzymes tagged with either His6-tag or His6-GST-tag were overexpressed in the *E. coli* BL21 strain but were insoluble (Additional file 1: Table S2). Thus, the three Ksh enzymes were further expressed as fusion protein with His6-MBP-tag. The His6-MBP fusion increased protein solubility as expected (Fig. 10e–g). The fusion proteins were purified by a Ni–NTA affinity chromatographic column (Fig. 11a–c) and their enzyme activities were evaluated by TLC (Fig. 11d). As shown in Lanes 2 and 6 in Fig. 11d, both KshA226 and KshA395 alone exhibited no activity within 20 h for 9α-hydroxylation of ADD. When in combination with KshB122 within 20 h, KshA226 and KshA395 started to slowly convert ADD to HSA via a transient intermediate, i.e., 9OH-ADD. By comparing the TLC image density in Fig. 11d, the conversion rates were estimated to be approximately 10% and 15% for the Ksh226- and Ksh395-mediated reactions, respectively.

Conversely, KshA226 and Ksh395 exhibited distinct specificity for the 9α-hydroxylation of 4-AD. Even in combination with KshB122, KshA226 exhibited no activity for 4-AD, while KshA395 exhibited a relatively high activity for the 9α-hydroxylation of 4-AD (Fig. 11e and Table 2). This observation was further confirmed by HPLC analysis. As revealed in Fig. 11f, KshA395/KshB122 significantly converted 4-AD to 9OH-AD within 40 h. The conversion of 4-AD to 9OH-AD by KshA395/KshB122 was monitored for 40 h (Fig. 11g), and the results indicated that KshA395/KshB122 had significant activity within 40 h, achieving conversion rate greater than 80%.

## Conclusion and discussion

In this work, *Mycobacterium neoaurum* HGMS2 strain with specific substrate was isolated and identified from soil samples, and 16S rRNA screening was performed to target *Mycobacterium* strains. *M. neoaurum* HGMS2 could accumulated 4-AD more efficiently than *M. neoaurum* NRRL B-3805. Based on comparative genomic analysis, the genomic features of the HGMS2 strain were closer to those of *M. neoaurum* NRRL B-3805 than other mycobacteria. The genome of the HGMS2 strain shared 88.1% nucleotide identity with B-3805. Using the B-3805 genome as reference, HGMS2 genome contained fewer nonsynonymous mutations than the genomes of *M*. MCS, *M*. KMV and *M*. H37Rv strains.

Through genomic annotation, we identified key enzymes involved in the HGMS2 phytosterol-degrading pathway. These key enzymes were classified into five groups based on their functions and reaction steps. Group 1 contained two cholesterol oxidases and one 3β-hydroxyl-dehydrogenase (ChoM1, ChoM2 and Hsd). Group 2 contained two monooxygenases (Mon164 and Mon197). Group 3 contained a set of fatty acid β-oxidoreductases. Group 4 contained one 3-ketosteroid-1,2-dehydrogenase (KstD211). Group 5 contained three 3-ketosteroid-9α-hydroxylases (KshA226, KshA395 and KshB122). Although many key enzyme genes were sporadically distributed in the HGMS2 genome, a gene cluster encoding KshB122, Mon164, Kstd211, KshA226 and fatty acid β-oxidoreductases constituted one integrated metabolic pathway. This pathway could completely degrade β-sitosterol via the 9.10-secosteroid intermediate once phytosterol substrates were oxidized and translocated into HGMS2 cells.

We prepared the recombinant forms of these key enzymes except for a set of fatty acid β-oxidoreductases in group 3 and evaluated their activities in vitro. Although these key enzymes are highly similar to those in other bacteria (Additional file 1: Figures S4–S10), they exhibited distinct activity compared with previous reported enzymes (Table 2). Our enzymatic data revealed that KshA226/KshB122 had no activity for either 4-AD or ADD, while KstD211 had weak activity. Such weak activity of KstD211 could enable the HGMS2 strain to accumulate 4-AD, accompanied by a small fraction of ADD during β-sitosterol fermentations. However, HGMS2 still enabled the production of a small fraction of 9OH-AD, as we identified that another 3-ketosteroid-9α-hydroxylase, i.e., KshA395, was active for both 4-AD and ADD. Therefore, our results suggested that KstD211 and KshA395 were the cause for the occurrence of ADD, 9OH-AD and HSA during prolonged β-sitosterol fermentation, where HSA would be readily metabolized to CO_2_ and H_2_O. The presence of active KstD211 and KshA395 not only reduced the yield of 4-AD but also made the purification of 4-AD more difficult.

Currently, the balance between the accumulation of 4-AD and the inhibition of 4-AD degradation during β-sitosterol fermentation of all industrial strains can be administered using vegetable oil-based media; at a practical level it is difficult to control the balance for a prolonged fermentation process, which often results in unstable conversion yield [6, 47]. This drawback is worsened when phytosterol fermentation is conducted in aqueous media, which is now in high demand for industrial application [48, 49]. Compared with the reference strains, HGMS2 had fewer genes for KstD and KshA isozymes, and is therefore a potential strain for gene manipulation. Furthermore, HGMS2 produced less 9OH-AD (Fig. 1). To block the ability of HGMS2 to generate ADD, 9OH-AD and HSA during β-sitosterol fermentations, the two enzymes for the formation of the 9,10-secosteroid intermediate can be completely deactivated. In an HGMS2 mutant in which *kstd211* and *kshA395* genes are knocked out the yield of AD, ADD and HSA is completely abolished (*Li *et al*., unpublished data*). In perspective, the HGMS2 strain also provides a new model to generate ADD-, 9OH-AD- and BA- producing strains for industrial application via gene engineering. ADD, 9OH-AD and BA are also important starting materials for the synthesis of advanced steroid medicines. It has proved feasible to use engineered bacteria to produce ADD, 9OH-AD and BA using β-sitosterol [50–52]. However, the yields of these bacterial strains are still very low and lack industrial applicability compared with mycobacterial transformation of β-sitosterol to 4-AD.

Although we partially identified and characterized the set of genes with products that are involved in HGMS2 phytosterol degradation, their regulation remains poorly understood. One of the next focuses should be on profiling the genome-wide response on phytosterol in HGMS2 grown with or without phytosterol, compared with other important steroid-producing strains.

## Supplementary information

**Additional file 1.** Additional tables and figures.

**Additional file 2: Table S3.** Comparison of genomes between *Mycobacterium neoaurum* HGMS2 and *M. neoaurum* NRRL B-3805

**Additional file 3: Figure S3.** Mapping of Mycobacterium neoaurum HGMS2 pathways

**Additional file 4: Table S6.** Animal pathogen analysis against PHI

**Additional file 5: Table S7.** Animal pathogen analysis against VFDB

**Additional file 6: Table S8.** Animal pathogen analysis against ARDB

## Data Availability

The *M. neoaurum* HGMS2 strain was deposited at China Center for Type Culture Collection (CCTCC NO: M2012522). This complete genome project was deposited in GenBank under accession number CP031414.1 and in BioProject under accession number PRJNA483955.

## Abbreviations

4-AD: 4-Androstene-3,17-dione
ADD: 1,4-Androstene-3,17-dione
9OH-AD: 9-Hydroxyl-4-androstene-3,17-dione
BA: 21-Hydroxy-20-methylpregn-4-en-3-one
BS: Bisulfite
Cho: Cholesterol oxidases
DCPIP: 2,6-Dichlorophenolindophenol
HSA: 3-Hydroxy-9,10-secoandrost-1,3,5(10)-triene-9,17-dione
Ksh: 3-Ketosteroid-9α-hydroxylase
KstD: 3-Ketosteroid-1,2-dehydrogenase
Mon: Monooxygenase
PMS: Phenazine methosulfate
