# Supplementary material for: Whole-genome and enzymatic analyses of an androstenedione-producing Mycobacterium strain with residual phytosterol-degrading pathways
Source: Microb Cell Fact. 2020 Oct 2;19:187. doi: 10.1186/s12934-020-01442-w (PMC7532642; doi:10.1186/s12934-020-01442-w)
Supplement: Supplementary file 1 — Additional file 1. Additional tables and figures. [file 12934_2020_1442_MOESM1_ESM.pdf]

Additional file 1: Additional tables and figures.

Whole-genome and Enzymatic Analyses of an Androstenedione-producing *Mycobacterium* Strain with Residual Phytosterol-degrading Pathways

Hongwei Wang[1], Shikui Song[1], Fei Peng[1], Fei Yang[1], Tian Chen[1], Xin Li[1], Xiyao Cheng[1,2], Yijun He[3], Yongqi Huang[1] and Zhengding Su[1,2,*]

[1]*Key Laboratory of Industrial Fermentation (Ministry of Education), Hubei Key Laboratory of Industrial Microbiology, National "111" Center for Cellular Regulation and Molecular Pharmaceutics, Hubei University of Technology, Wuhan, 430068, China*

[2]*Wuhan Amersino Biodevelop Inc., B1-Building, Biolake Park, Wuhan, Hubei, 430075, China*

[3]*Hubei Goto Biotech Inc. No. 1 Baiguoshu Road, Shuidu Industrial Park, Danjiangkou, Hubei, 442700, China*

[*]**Corresponding Author**: Zhengding Su, Email: zhengdingsu@hbut.edu.cn, Tel: 86-156-23901978

Keywords: 1,4-androstene-3,17-dione (ADD); 3-hydroxy-9,10-secoandrost-1,3,5(10)-triene-9,17-dione (HSA); 3-ketosteroid-1,2-dehydrogenase (Ksh); 3-ketosteroid-9α-hydroxylase; 4-androstene-3,17-dione (4-AD); 9-hydroxyl-4-androstene-3,17-dione (9OH-AD); 21-hydroxy-20-methylpregn-4-en-3-one (BA); biotransformation; cholesterol oxidases (Cho); monooxygenase (MO); *Mycobacterium sp*. strain.

Methods, Results and Discussion

Procedure of genomic sequencing of Mycobacterium neoaurum HGMS2 strain

Figure S1a outlines a genomic sequencing procedure for *Mycobacterium neoaurum* HGMS2. **Figures S1b-1c** show statistics of qualified reads.

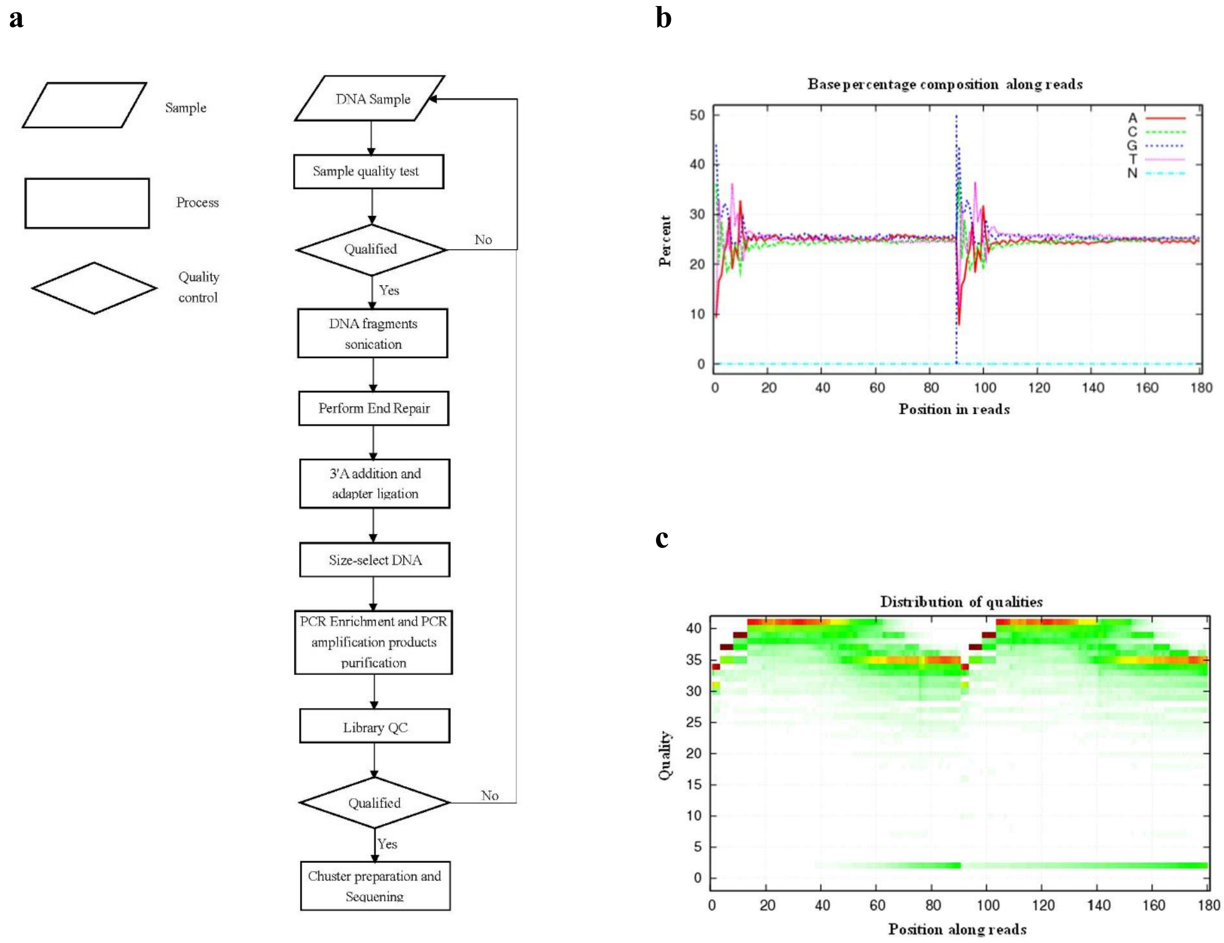

Figure S1. Procedure and quality control test of genomic sequencing for *Mycobacterium neoaurum* HGMS2. a. PacBio experimental process. **b**. Base percentage composition along reads. 1-90 bp represent the base position of read 1 and 91-180 bp represent the base position of read 2.

c. Quality distribution along position reads. For each sample lane during sequencing, the less spots below 20 along quality parameter are, the better quality of sequencing is indicated.

Primers and constructed plasmids for the preparation of soluble key enzymes

Table S1. Primers used to amplify the key genes in HGMS2 phytosterol-degrading pathway by PCR

Gene	Length (bp)	Primer	Sequence （5'→ 3'）
ChoM1	1746	Forward	CGCCGC**AGATCT**ATGAAGCCTGACTATGAC (BglII)
		Reverse	CGCCGC**GAATTC**TTACGCCGAGGGCGACAG (EcoRI)
ChoM2	1617	Forward	CGCGA**AGATCT**ATGCTGACAAGACGGCGG (BglII)
		Reverse	CCG**GAATTC**CTAACGCCGGCCTGAGATG (EcoRI)
Hsd	1101	Forward	CGC**GGATCC**ATGGGTGACCCAACTTTG (BamHI)
		Reverse	CGCCGC**GAATTC**TCAGCTCTTCGGTGCTGC (EcoRI)
MO164	1251	Forward	CGCCGC**GGATCC**ATGCCCAGCCCCAACCTG(BamHI)
		Reverse	CGCCGC**GAATTCC**TAGCTCGAGGCGCCGGC (EcoRI)
MO197	1272	Forward	CGCCGC**AGATCT**ATGACCGACACCGCCCAC (BglII)
		Reverse	CGCCGC**CTCGAG**CTAGTGGTGCACAGCCTG (XhoI)
KstD211	1689	Forward	GTA**GGATCC**ATGACTGAACAGGACTAC (BamHI)
		Reverse	GCA**GAATTC**TCAGGCCTTTCCAGCGAG (EcoRI)
KshA226	1185	Forward	CCG**GGATCC**GTGACTACCGAGACAGCC (BamHI)
		Reverse	CCG**CTCGAG**TCAGCTCGGCTGCGCGGA (XhoI)
KshA395	1146	Forward	CAGTA**GGATCC**ATGACCGATATCCGCGA (BamHI)
		Reverse	GCA**GAATTC**TCACCGTTGCGCGGTGGT (EcoRI)
KshB122	1053	Forward	GTA**GGATCC**GTGACGGAGGAACCGCTC (BamHI)
		Reverse	GCA**GAATTC**CTATTCGTCGTAGGTGAC (EcoRI)

Notes: restriction enzyme sites introduced in each primer are underlined and highlighted in bold.

Table S2. Summary of protein expression plasmids and solubility

Protein	Fusion tag	Vector	Construct	Solubility	SDS-PAGE
ChoM1	His6	pSZD	pSZD-ChoM1	tiny	**Figure S11**
	His6-GST	pGST	pGST-ChoM1	No	**Figure S12**
	His6-MBP	pMBP	pMBP-ChoM1	Yes	
ChoM2	His6	pSZD	pSZD-ChoM2	NT	
	His6-GST	pGST	pGST-ChoM2	NT	
	His6-MBP	pMBP	pMBP-ChoM2	Yes	
Hsd	His6	pSZD	pSZD-Hsd	No	
	His6-GST	pGST	pGST-Hsd	No	**Figure S13**
	His6-MBP	pMBP	pMBP-Hsd	Yes	
Mon164	His6	pSZD	pSZD-Mon164	Yes	
	His6-GST	pGST	pGST-Mon164	NT	
	His6-MBP	pMBP	pMBP-Mon164	NT	
Mon197	His6	pSZD	pSZD-Mon197	No	**Figure S14**
	His6-GST	pGST	pGST-Mon197	No	
	His6-MBP	pMBP	pMBP-Mon197	Yes	
KstD211	His6	pSZD	pSZD-KstD211	Yes	
	His6-GST	pGST	pGST-KstD211	NT	
	His6-MBP	pMBP	pMBP-KstD211	NT	
KshA226	His6	pSZD	pSZD-KshA226	No	**Figure S15**
	His6-GST	pGST	pGST-KshA226	No	
	His6-MBP	pMBP	pMBP-KshA226	Yes	
KshA395	His6	pSZD	pSZD-KshA395	No	**Figure S16**
	His6-GST	pGST	pGST-KshA395	No	
	His6-MBP	pMBP	pMBP-KshA395	Yes	
KshB122	His6	pSZD	pSZD-KshB122	NO	**Figure S17**
	His6-GST	pGST	pGST-KshB122	No	
	His6-MBP	pMBP	pMBP-KshB122	Yes	

Note: NT, not tested

Mass spectra of steroid intermediates

The LC/MS mass spectra of HPLC fractions were obtained under isocratic or gradient elution conditions, with acetonitrile:water (1:1) as the mobile phase. Small percentages of formic acid were added to the water mobile phase in some experiments to check the effects of the additives on the sensitivities and the mass spectra of the steroid compounds. After choosing the best conditions, detection limit was assessed for each fraction using selected ion monitoring of the most abundant ions in the spectra. The results were shown in **Figure S2**, identifying expected HPLC fractions.

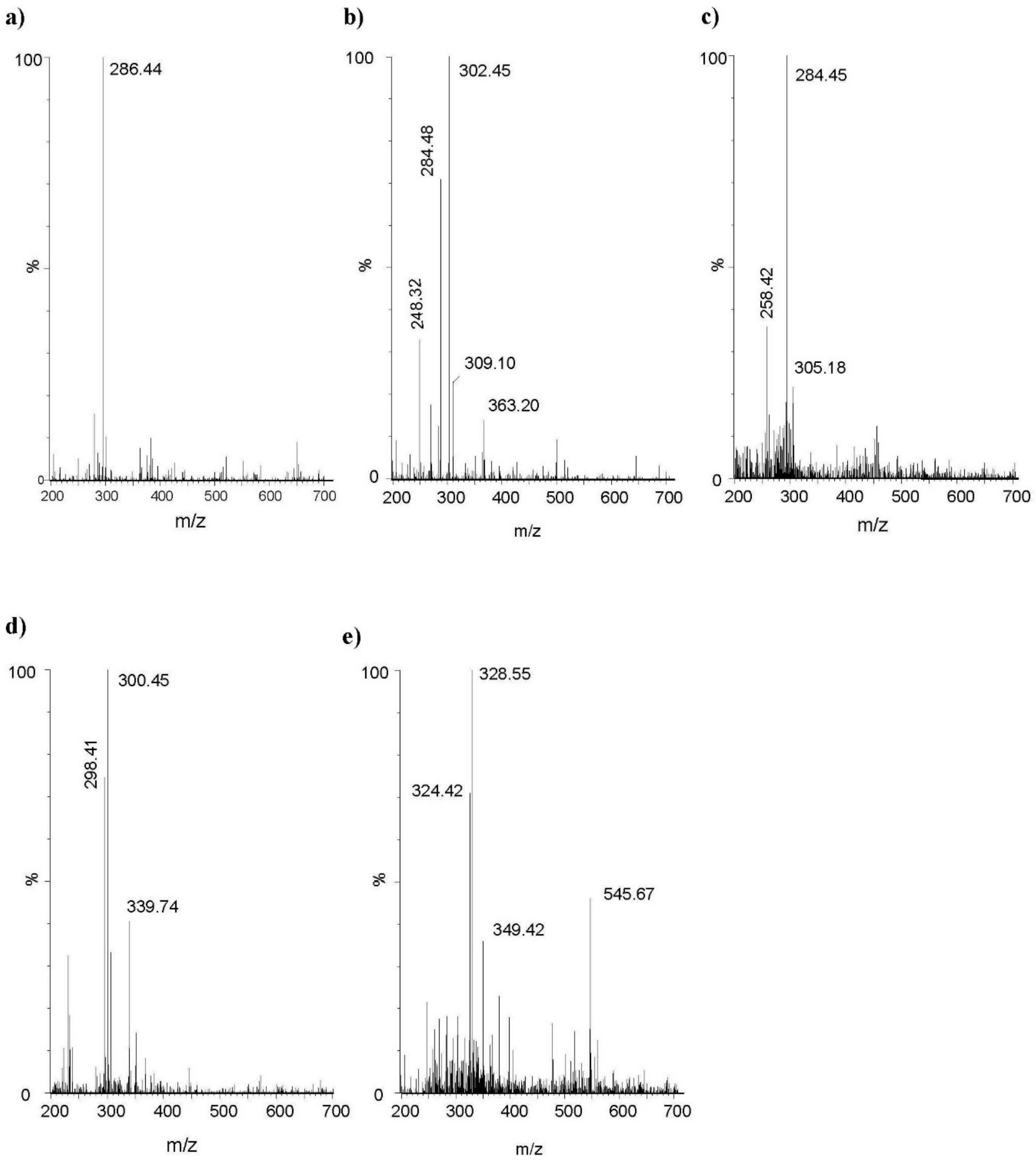

Figure S2. Mass spectra of metabolites during biotransformation of β-sitosterol by *Mycobacterium neoaurum* HGMS2. a. 4-AD; **b**. ADD; **c**. 9OH-AD; **d**. HSA; **e**. BA.

Comparative analysis of HGMS2 and B-3805 genomics

The statistics of HGMS2 genome was summarized in **Table S3**, in comparison with B-3805.

Please note that **Table S3** was uploaded as a separated electronic file.

Classification of Gene functions in HGMS2

Table S4. Gene functions of HGMS2

Class	Functions	amounts
1	RNA processing and modification	1
2	Chromatin structure and dynamics	1
3	Energy production and conversion	232
4	cell division, chromosome partitioning	26
5	Amino acid transport and metabolism	322
6	nucleotide transport and metabolism	71
7	Carbohydrate transport and metabolism	187
8	Coenzyme transport and metabolism	134
9	Lipid transport and metabolism	291
10	Translation, ribosomal structure and biogenesis	153
11	Transcription	225
12	Replication, recombination and repair	132
13	Cell wall/membrane/envelope biogenesis	119
14	Cell motility	5
15	posttranslational modification, protein turnover, chaperones	98
16	inorganic ion transport and metabolism	208

17	Secondary metabolites biosynthesis, transport and catabolism	2x3
18	General function prediction only	485
19	Function unknown	199
20	Signal transduction mechanism	103
21	Intracellular trafficking, secretion, and vesicular transport	27
22	Defense mechanisms	27

Mapping of HGMS2 metabolic pathways and statistics of core genes and pangenes

We aligned HGMS genes with databases to obtain their corresponding annotations. To ensure the biological meaning, the highest quality alignment result was chosen as gene annotation. Function annotation was completed by blasting genes with different databases, including the following nine databases:

(1) Kyoto Encyclopedia of Genes and Genomes (KEGG, Version 59) [1][2][3].

(2) Cluster of Orthologous Groups of proteins (COG, Version:20090331) [4][5].

(3) SwissProt (Version 201206) [6].

(4) NR (Version 20121005).

(5) Gene Ontology (GO, Version 1.419) [7].

(6) Pathogen Host Interactions (PHI, Version:3.2)[8].

(7) Antibiotic Resistance Genes Database (ARDB, Version 1.1)[9].

(8) Virulence Factors of Pathogenic Bacteria (VFDB, Version 20130128)[10].

(9) Carbohydrate-Active enZYmes Database (CAZy, Version:20101214) [11].

Detailed metabolic pathways in *M.* HGMS2 were mapped by comparing with the databases within and outside of *Mycobacterium* group and were summarized in **Figure S3**. Please note that **Figure S3** was provided by a separated electronic ZIP file.

The genomes of different bacteria (4 samples) were compared. The genes shared by all of the bacteria were core genes that most of the genes were genes necessary for growth, and the genes were special genes that they were contained only by one of the bacteria. Difference special genes were important for the detection of the functional differences and similarities between samples, and provided molecular evidences for the phenotype differences and similarities. Core-Pan gene analysis were carried out with the genome of NRRL-B3805 as a reference and the genomes of KMS, MCS, H37Rv and HGMS2 as queries. Statistics of their core-pan genes were summarized in **Table S5**.

Table S5 Statistics of core-pan genes

**Core-genome**		**Pan-genome**	
Gene Number	Total size (aa)	Gene Number	Total size (aa)
3,327	1,160,343	6,921	2,127,292

Animal pathogenic annotation of the HGMS strain were compared against the Pathogen Host Interaction (PHI) database, Virulence Factors database (VFDB) and Antibiotic Resistance Genes database (ARDB). The results were listed in **Tables S6-S8**, respectively, indicating that HGMS2 was nonvirulent.

Please note that **Table S6-S8** were provided by individual electronic files.

Comparison of KstD211 with other bacterial KstDs

The amino acid sequence of KstD211 was aligned with the KstD enzymes from bacteria within and outside of *Mycobacterium* sp. genus, including *Mycobacterium* sp. DSM1381, *Mycobacterium* sp. MC^2 155, *A. simplex*, *Rhodococcus* sp. SQ1 and etc. As shown in **Figure S4**, KstD211 is highly homologous in 75%, 73%, 65% and 60%, respectively, to those reported KstDs. Nevertheless, KstD211 was quite similar to those from NRRL-B-3805, KMS, H37Rv and MCS strains (**Figure S5**).

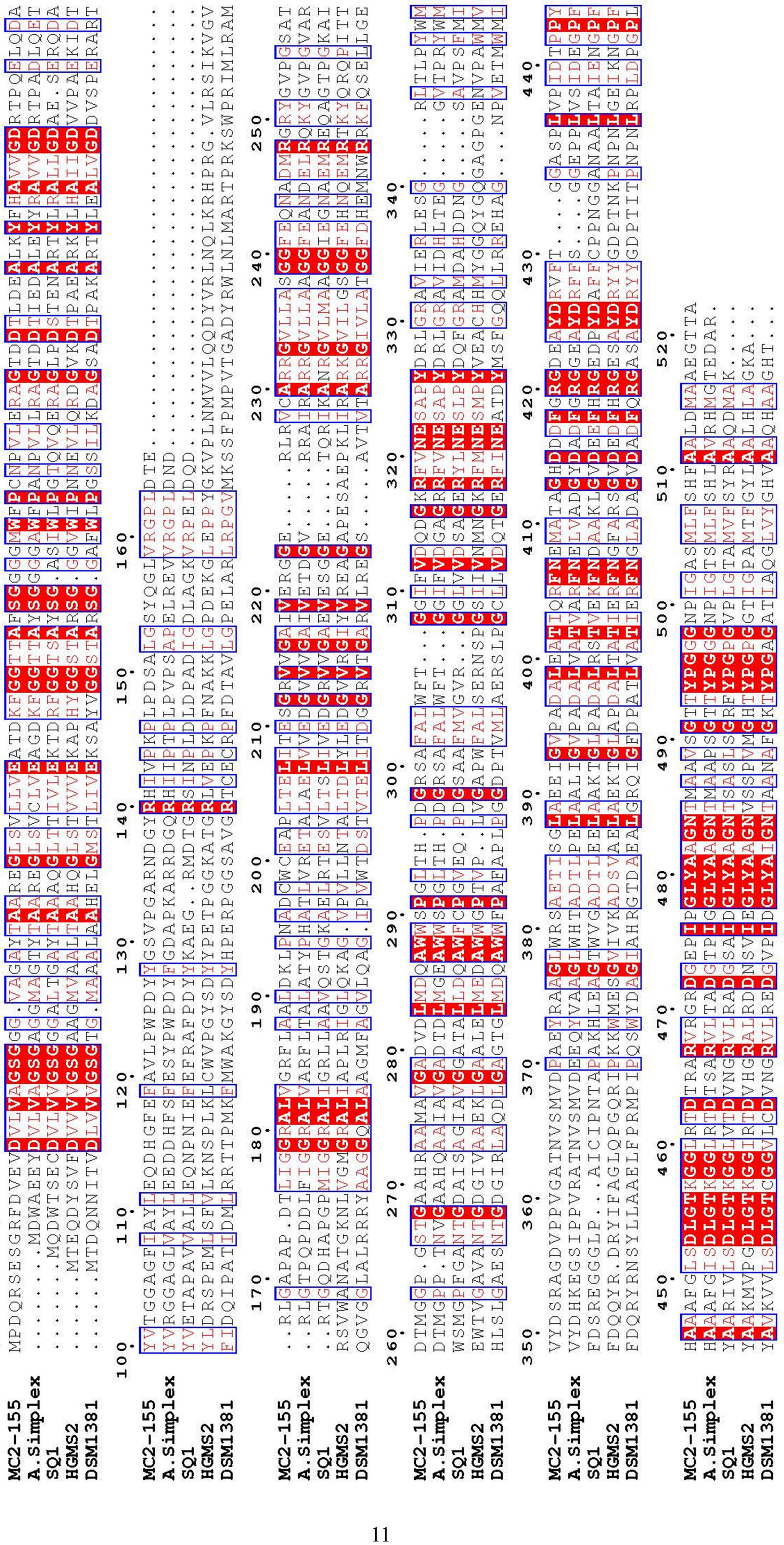

Figure S4. Amino acid alignment of KstD211 with other KstD enzymes. DSM1381: *Mycobacterium sp.* DSM1381; MC^2 155: *Mycobacterium sp.* MC^2 155; *A. simplex*: *Arthrobacter simplex* and SQ1: *Rhodococcus erythropolis* SQ1. Solid and open frames indicated identical and similar residues, respectively.

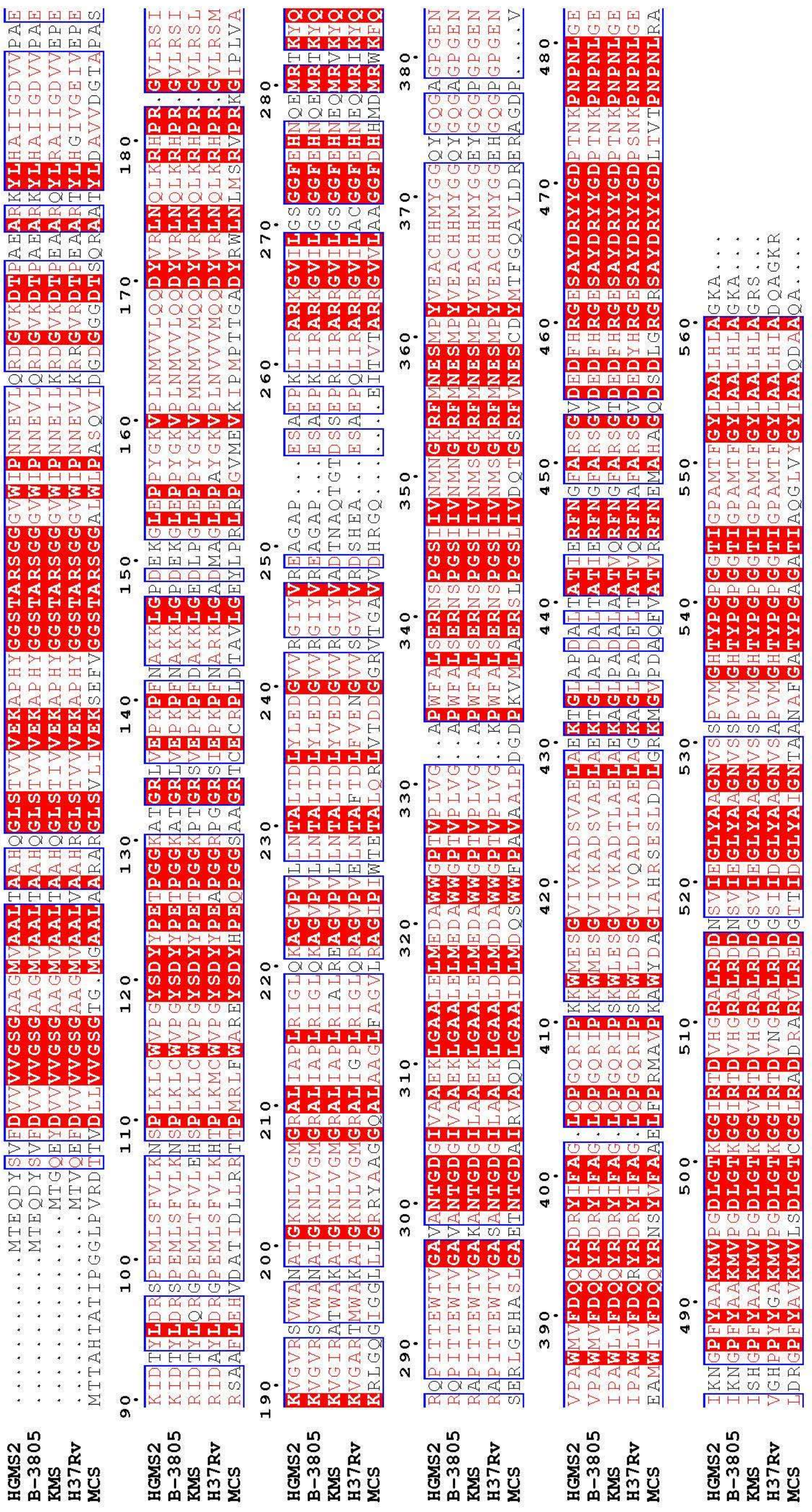

Figure S5. Amino acid Alignment of KstD211 from HGMS2 with other mycobacterial KstD enzymes. B-3805: *Mycobacterium sp.* NRRL B-3805; KMS: *Mycobacterium sp.* KMS; H37Rv: *Mycobacterium sp.* H37Rv and MCS: *Mycobacterium sp.* MCS. Solid and open frames indicated identical and similar residues, respectively.

Comparison of ChoM1 and ChoM2 with other bacterial ChoM

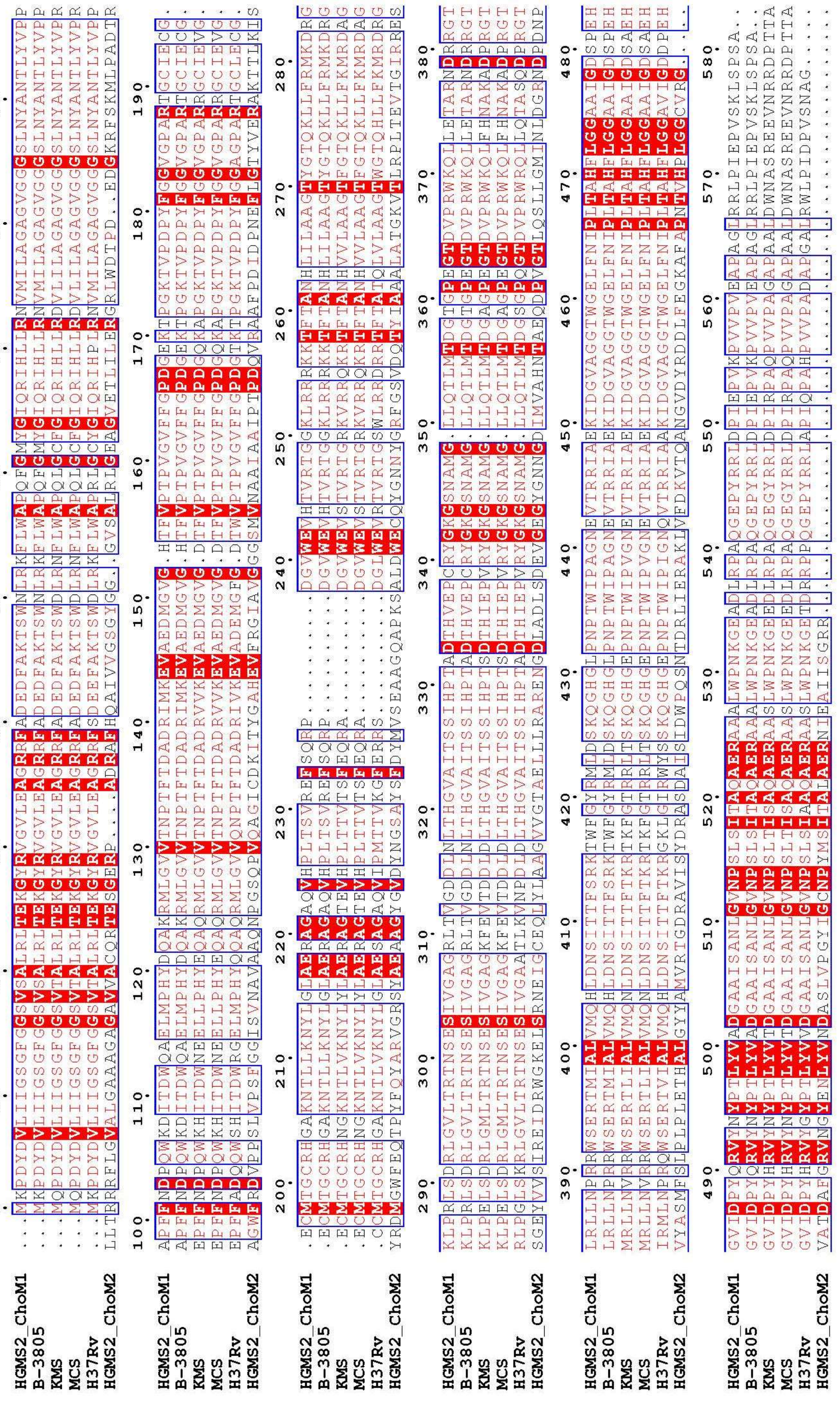
HGMS2_ChoM1
B-3805
KMS
MCS
H37Rv
HGMS2_ChoM2

Figure S6. Amino acid Alignment of ChoM1 and ChoM2 from HGMS2 with other mycobacterial ChoM enzymes. B-3805, *Mycobacterium* sp. NRRL B-3805; KMS, *Mycobacterium* sp. KMS; H37Rv, *Mycobacterium* sp. H37Rv and MCS, *Mycobacterium* sp. MCS. Filled and opened boxes indicated identical and similar residues, respectively.

Comparison of Hsd with other bacterial Hsd

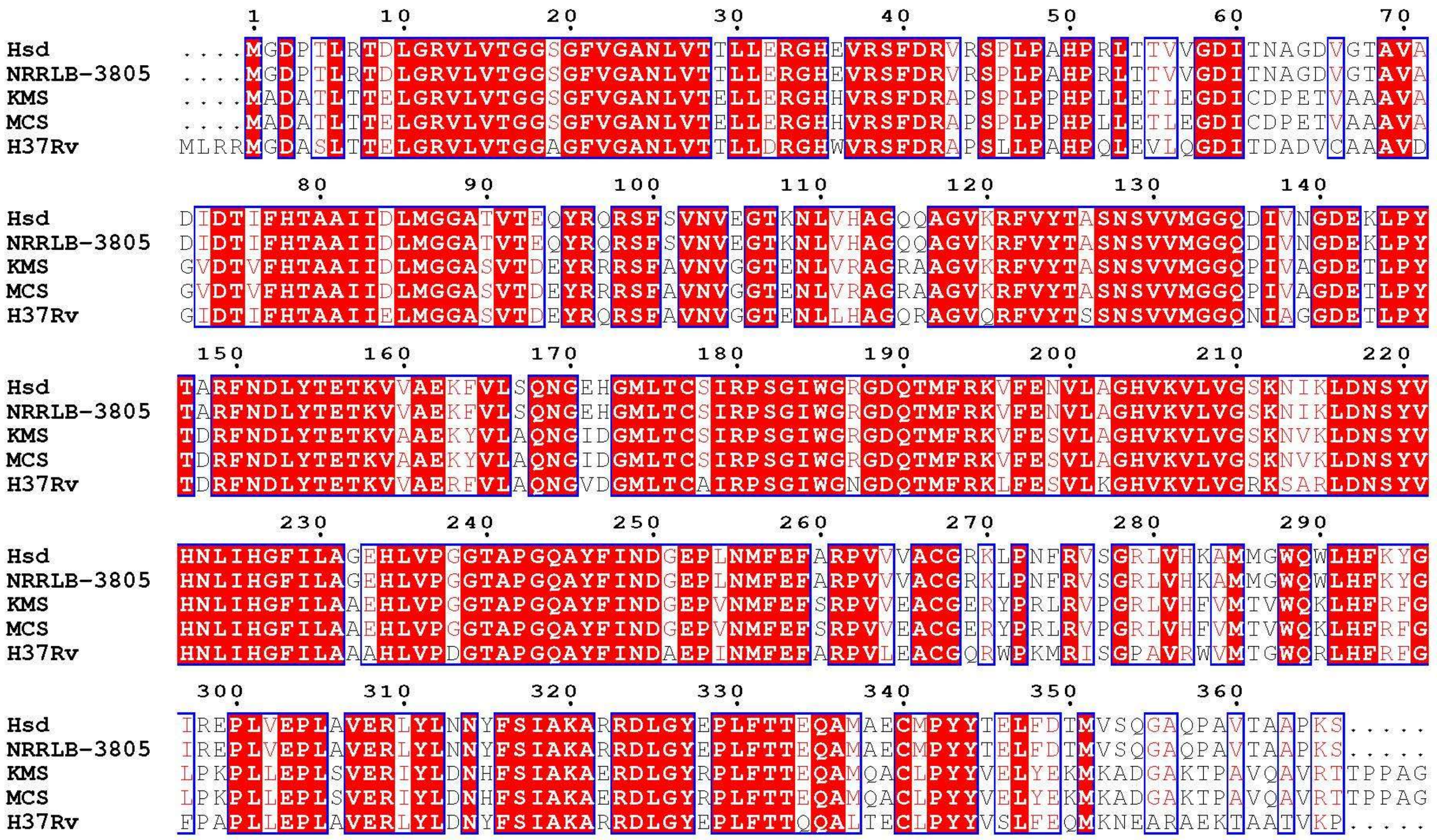
1 10 20 30 40 50 60 70
Hsd ....MGDPTLRTDLGRVLVTGGSGFVGANLVTTLLERGHEVRSFDRVRSPLPAHPRLTTVVGDITNAGDVGTAVA
NRRLB-3805 ....MGDPTLRTDLGRVLVTGGSGFVGANLVTTLLERGHEVRSFDRVRSPLPAHPRLTTVVGDITNAGDVGTAVA
KMS ....MADATLTTELGRVLVTGGSGFVGANLVTELLERGHHVRSFDRAPSPLPPHPLLETLEGDICDPETVAAAVA
MCS ....MADATLTTELGRVLVTGGSGFVGANLVTELLERGHHVRSFDRAPSPLPPHPLLETLEGDICDPETVAAAVA
H37Rv MLRRMGDASLTTELGRVLVTGGAGFVGANLVTTLLDRGHWVRSFDRAPSLLPAHPQLEVLQGDITDADVCAAAVD
80 90 100 110 120 130 140
Hsd DIDTIFHTAAIIDLMGGATVTEQYRQRSFSVNVEGTKNLVHAGQQAGVKRFVYTASNSVVMGGQDIVNGDEKLPY
NRRLB-3805 DIDTIFHTAAIIDLMGGATVTEQYRQRSFSVNVEGTKNLVHAGQQAGVKRFVYTASNSVVMGGQDIVNGDEKLPY
KMS GVDTVFHTAAIIDLMGGASVTDEYRRRSFAVNVGGTENLVRAGRAAGVKRFVYTASNSVVMGGQPIVAGDETLPY
MCS GVDTVFHTAAIIDLMGGASVTDEYRRRSFAVNVGGTENLVRAGRAAGVKRFVYTASNSVVMGGQPIVAGDETLPY
H37Rv GIDTIFHTAAIIELMGGASVTDEYRQRSFAVNVGGTENLLHAGQRAGVQRFVYTSSNSVVMGGQNIAGGDETLPY
150 160 170 180 190 200 210 220
Hsd TARFNDLYTETKVVAEKFVLSQNGEHGMLTCSIRPSGIWGRGDQTMFRKVFENVLAGHVKVLVGSKNIKLDNSYV
NRRLB-3805 TARFNDLYTETKVVAEKFVLSQNGEHGMLTCSIRPSGIWGRGDQTMFRKVFENVLAGHVKVLVGSKNIKLDNSYV
KMS TDRFNDLYTETKVAAEKYVLAQNGIDGMLTCSIRPSGIWGRGDQTMFRKVFESVLAGHVKVLVGSKNVKLDNSYV
MCS TDRFNDLYTETKVAAEKYVLAQNGIDGMLTCSIRPSGIWGRGDQTMFRKVFESVLAGHVKVLVGSKNVKLDNSYV
H37Rv TDRFNDLYTETKVVAERFVLAQNGVDGMLTCAIRPSGIWGNGDQTMFRKLFESVLKGHVKVLVGRKSARLDNSYV
230 240 250 260 270 280 290
Hsd HNLIHGFILAGEHLVPGGTAPGQAYFINDGEPLNMFEFARPVVVACGRKLPNFRVSGRLVHKAMMGWQWLHFKYG
NRRLB-3805 HNLIHGFILAGEHLVPGGTAPGQAYFINDGEPLNMFEFARPVVVACGRKLPNFRVSGRLVHKAMMGWQWLHFKYG
KMS HNLIHGFILAAEHLVPGGTAPGQAYFINDGEPVNMFEFSRPVVEACGERYPRLRVPGRLVHFVMTVWQKLHFRFG
MCS HNLIHGFILAAEHLVPGGTAPGQAYFINDGEPVNMFEFSRPVVEACGERYPRLRVPGRLVHFVMTVWQKLHFRFG
H37Rv HNLIHGFILAAAHLVPDGTAPGQAYFINDAEPINMFEFARPVLEACGQRWPKMRISGPAVRWVMTGWQRLHFRFG
300 310 320 330 340 350 360
Hsd IREPLVEPLAVERLYLNNYFSIAKARRDLGYEPLFTTEQAMAECMPYYTELFDTMVSQGAQPAVTAAPKS.....
NRRLB-3805 IREPLVEPLAVERLYLNNYFSIAKARRDLGYEPLFTTEQAMAECMPYYTELFDTMVSQGAQPAVTAAPKS.....
KMS LPKPLLEPLSVERIYLDNHFSIAKAERDLGYRPLFTTEQAMQACLPYYVELYEKMKADGAKTPAVQAVRTTPPAG
MCS LPKPLLEPLSVERIYLDNHFSIAKAERDLGYRPLFTTEQAMQACLPYYVELYEKMKADGAKTPAVQAVRTTPPAG
H37Rv FPAPLLEPLAVERLYLDNYFSIAKARRDLGYEPLFTTQQALTECLPYYVSLFEQMKNEARAEKTAATVKP.....

Figure S7. Amino acid Alignment of Hsd from HGMS2 with other mycobacterial Hsd enzymes. B-3805, *Mycobacterium* sp. NRRL B-3805; KMS, *Mycobacterium* sp. KMS; H37Rv, *Mycobacterium* sp. H37Rv and MCS, *Mycobacterium* sp. MCS. Filled and opened boxes indicated identical and similar residues, respectively.

Comparison of Mon164 and Mon197 with other bacterial Mons

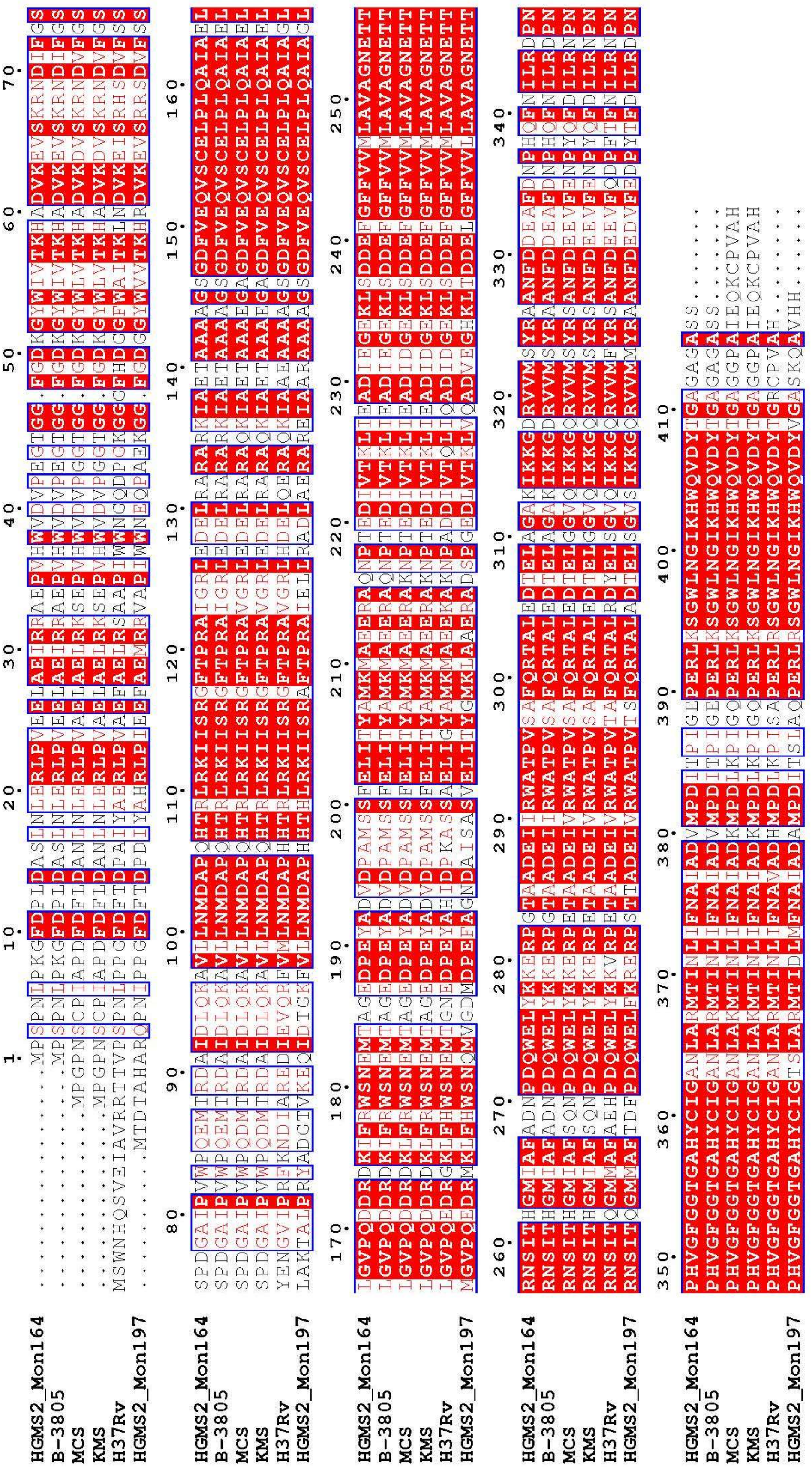
HGMS2_Mon164
B-3805
MCS
KMS
H37Rv
HGMS2_Mon197

Figure S8. Amino acid Alignment of Mon164 from HGMS2 with other mycobacterial Mons enzymes. B-3805, *Mycobacterium* sp. NRRL B-3805; KMS, *Mycobacterium* sp. KMS; H37Rv, *Mycobacterium* sp. H37Rv and MCS, *Mycobacterium* sp. MCS. Filled and opened boxes indicated identical and similar residues, respectively.

Comparison of KshA226 and Ksh395 with other bacterial KshAs

```
                1        10        20        30        40        50        60        70        80
HGMS2_KshA395.  ......MTDIREIDAGVAMTRFARGWHCLGLAETFRDGRPHGIEAFGSKLVVFGDTGGALHVLDAYCRHMGGDLSRGSVKDDNLACPFH
B-3805_KshA     ......MTDIREIDAGVAMTRFARGWHCLGLAETFRDGRPHGIEAFGSKLVVFGDTGGALHVLDAYCRHMGGDLSRGSVKDDNLACPFH
HGMS2_KshA226   ..VTTETAGIREIDTGDLPDRYARGWHCLGPVKDYLDGKPHGVEIFDTMLVVFADSEGELKVLDGYCRHMGGNLAQGTIKGDTVACPFH
KMS_KshA        MSTDTAHSGIREIDTGTLPDRYARGWHCLGPVNDYLDGEPHSVEAFGTKLVVFADSKGDVKILDGYCRHMGGDLSQGTIKGDEVACPFH
H37Rv_KshA.     MSTDTSGVGVREIDAGALPTRYARGWHCLGVAKDYLEGKPHGVEAFGTKLVVFADSHGDLKVLDGYCRHMGGDLSEGTVKGDEVACPFH
MCS_KshA        ..MTSDIDEVRQIEADAAPTRFARGWHCLGLTRDFGDGKPHTVNAFGQKLVVFRSGDGRINVLDGYCRHMGGDLSQGEVKGNEIACPFH

                    90       100       110       120       130       140       150       160       170
HGMS2_KshA395.  DWRWRADGRCALVPYAKRTPRLARTRAWETREVNGQLLVWHDPEGSAPPAGLLPPTIEGYPEGRWSPWQWNSVLIEGSHCREIVDNNVD
B-3805_KshA     DWRWRADGRCALVPYAKRTPRLARTRAWETREVNGQLLVWHDPEGSAPPAGLLPPTIEGYPEGRWSPWQWNSVLIEGSHCREIVDNNVD
HGMS2_KshA226   DWRWGGDGKCKLVPYAKRTPRLARTRAWHTDVRGGLLFVWHDHEGNPPQPEVRIPEIPEFASDDWTDWRWNTMLIEGSNCREIIDNVTD
KMS_KshA        DWRWGGDGKCKLVPYAKRTPRLARTRAWTTDVRSGLLFVWHDHEGNPPPPEVRIPEIPEFASDEWTDWRWNSILIEGANCREIIDNVTD
H37Rv_KshA.     DWRWGGDGRCKLVPYARRTPRMARTRSWTTDVRSGLLFVWHDHEGNPPDPAVRIPEIPEAASDEWTDWRWNRILIEGSNCRDIIDNVTD
MCS_KshA        DWRWGGDGRCKQVPYSKRAPRLARTATWTTLEQDGMLFVWNDPERKPPPDDVTIPRIEGATSDEWTDWHWYTTVVDTN.CREVIDNVVD

                   180       190       200       210       220       230       240       250       260
HGMS2_KshA395.  MAHFFYIHHAYPTFFKNVIEGHTASQFMESKPRPDYIADPEKLWEGTYLRSEATYFGPAYMINWLHNDLAPGFTVEVALINCHYPVSHD
B-3805_KshA     MAHFFYIHHAYPTFFKNVIEGHTASQFMESKPRPDYIADPEKLWEGTYLRSEATYFGPAYMINWLHNDLAPGFTVEVALINCHYPVSHD
HGMS2_KshA226   MAHFFYIHYGLPTYFKNVFEGHIASQYLHNVGRPD.VNDLGTTYGEAHLDSEASYFGPSFMINWLHNNYG.GFKAESILINRHYPVSQD
KMS_KshA        MAHFFYIHFGLPTYFKNVFEGHIASQYLHNVGRPD.VNDMGTTYGEAHLDSEASYFGPSFMINWLHNNYG.GYKAESILINCHYPVTQD
H37Rv_KshA.     MAHFFYIHFGLPTYFKNVFEGHIASQYLHNVGRPD.VDDLGTSYGEAHLDSEASYFGPSFMINWLHNRYG.NYKSESILINCHYPVTQN
MCS_KshA        MAHFFYIHGSLPTHFKNIFEGHVATQYMNSAGRADLQPPEG..PRMLGTTSVASYHGPSFMIDDLTYHYEEADHHT.VLINCHYPIDAN

                     270       280       290       300       310       320       330       340       350
HGMS2_KshA395.  SFVLQWGVAVQQMPGLPADKAAKLAGAMSRSFGEGFMEDVEIWRHKTRIENPLLTEEDGAVYQHRRWYEQFYVDAADVTADMTDRFELE
B-3805_KshA     SFVLQWGVAVQQMPGLPADKAAKLAGAMSRSFGEGFMEDVEIWRHKTRIENPLLTEEDGAVYQHRRWYEQFYVDAADVTADMTDRFELE
HGMS2_KshA226   SFVLQWGVIVEKPKGLDEKTTDKLARVFTEGVSKGFLQDVEIWKHKTRIDNPLLVEEDGAVYQMRRWYQQFYVDVADVTPDMTDRFEME
KMS_KshA        SFVLQWGVIVEKPKGMDEKMTDKLARTFTDGVSKGFLQDVEIWKHKTRIDNPLLVEEDGAVYQLRRWYQQFYVDVADVTPEMTDRFEIE
H37Rv_KshA.     SFVLQWGVIVEKPKGMSEEMTDKLSRVFTEGVSKGFLQDVEIWKHKTRIDNPLLVEEDGAVYQLRRWYEQFYVDVADIKPEMVERFEIE
MCS_KshA        SFVLQYGIIVKKSEALPDDLAVQTAVSLGDFVKMGFEQDVEIWKNKARIDNPLLCEEDGPVYQLRRWYEQFYVDIADVTPDMVDRFEYE

                       360       370       380
HGMS2_KshA395.  IDTTHAYGIWAEEVAENLAGLAQAGGGTTAQR...........
B-3805_KshA     IDTTHAYGIWAEEVAENLAGLAQAGGGTTAQR...........
HGMS2_KshA226   VDTTIANEKWHVEVEENLKLQQDAAEQDAAEQGEPQKESAQPS
KMS_KshA        VDTTAANEYWNTEVQENLARREGEKAEQPTP............
H37Rv_KshA.     VDTKRANEFWNAEVEKNLKSREVSDDVPAEQH...........
MCS_KshA        VDTTRPREEWMKIVEANISARASATSAG...............
```

Figure S9. Amino acid Alignment of KshA395 and KshA226 from HGMS2 with other mycobacterial KshA enzymes. B-3805, *Mycobacterium* sp. NRRL B-3805; KMS, *Mycobacterium* sp. KMS; H37Rv, *Mycobacterium* sp. H37Rv and MCS, *Mycobacterium* sp. MCS. Filled and opened boxes indicated identical and similar residues, respectively.

Comparison of KshB122 with other bacterial KshBs

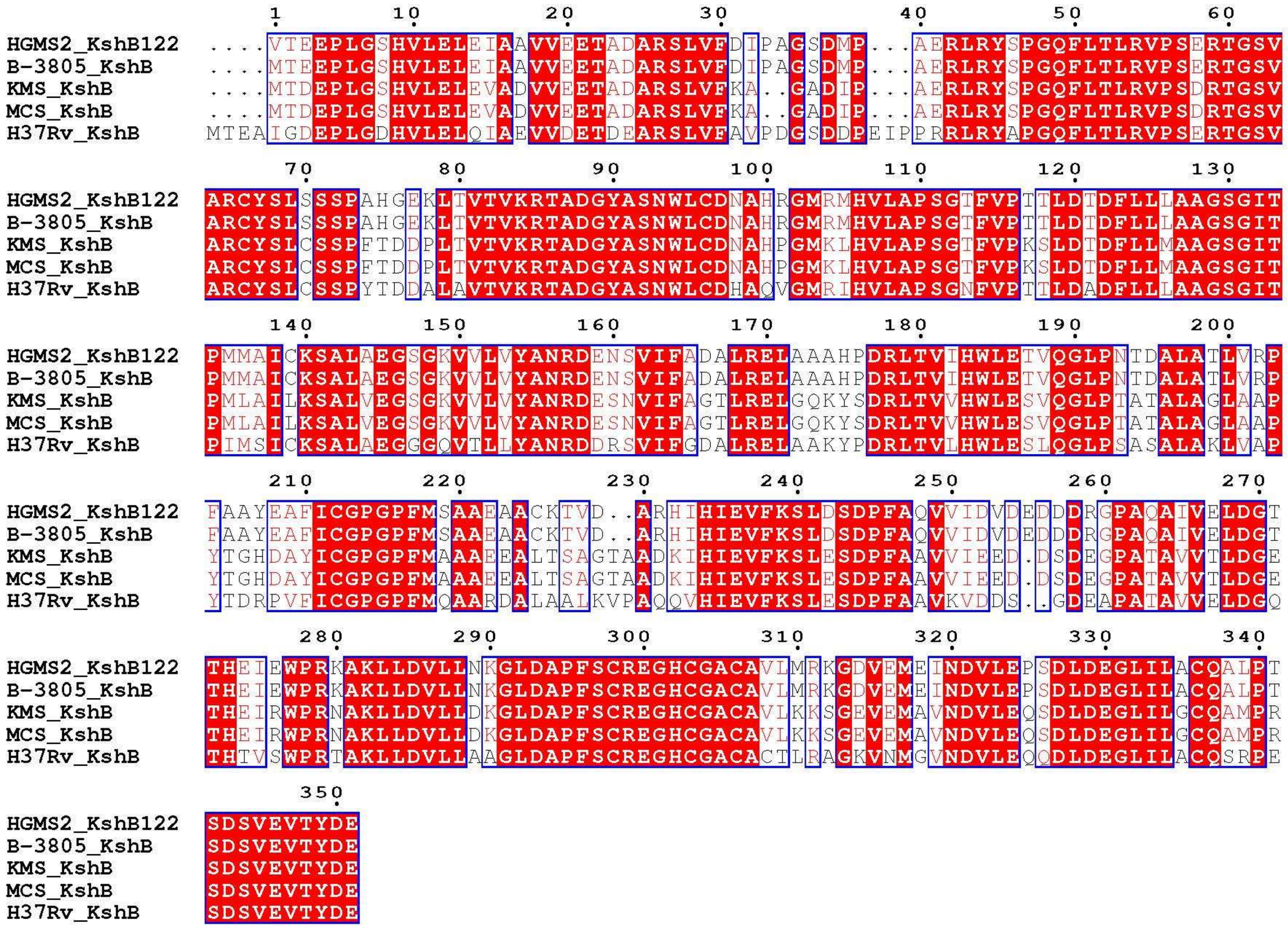
1 10 20 30 40 50 60
HGMS2_KshB122 ....VTEEPLGSHVLELEIAAVVEETADARSLVFDIPAGSDMP...AERLRYSPGQFLTLRVPSERTGSV
B-3805_KshB ....MTEEPLGSHVLELEIAAVVEETADARSLVFDIPAGSDMP...AERLRYSPGQFLTLRVPSERTGSV
KMS_KshB ....MTDEPLGSHVLELEVADVVEETADARSLVFKA..GADIP...AERLRYSPGQFLTLRVPSDRTGSV
MCS_KshB ....MTDEPLGSHVLELEVADVVEETADARSLVFKA..GADIP...AERLRYSPGQFLTLRVPSDRTGSV
H37Rv_KshB MTEAIGDEPLGDHVLELQIAEVVDETDEARSLVFAVPDGSDDPEIPPRRLRYAPGQFLTLRVPSERTGSV
70 80 90 100 110 120 130
HGMS2_KshB122 ARCYSLSSSPAHGEKLTVTVKRTADGYASNWLCDNAHRGMRMHVLAPSGTFVPTTLDTDFLLLAAGSGIT
B-3805_KshB ARCYSLSSSPAHGEKLTVTVKRTADGYASNWLCDNAHRGMRMHVLAPSGTFVPTTLDTDFLLLAAGSGIT
KMS_KshB ARCYSLCSSPFTDDPLTVTVKRTADGYASNWLCDNAHPGMKLHVLAPSGTFVPKSLDTDFLLMAAGSGIT
MCS_KshB ARCYSLCSSPFTDDPLTVTVKRTADGYASNWLCDNAHPGMKLHVLAPSGTFVPKSLDTDFLLMAAGSGIT
H37Rv_KshB ARCYSLCSSPYTDDALAVTVKRTADGYASNWLCDHAQVGMRIHVLAPSGNFVPTTLDADFLLLAAGSGIT
140 150 160 170 180 190 200
HGMS2_KshB122 PMMAICKSALAEGSGKVVLVYANRDENSVIFADALRELAAAHPDRLTVIHWLETVQGLPNTDALATLVRP
B-3805_KshB PMMAICKSALAEGSGKVVLVYANRDENSVIFADALRELAAAHPDRLTVIHWLETVQGLPNTDALATLVRP
KMS_KshB PMLAILKSALVEGSGKVVLVYANRDESNVIFAGTLRELGQKYSDRLTVVHWLESVQGLPTATALAGLAAP
MCS_KshB PMLAILKSALVEGSGKVVLVYANRDESNVIFAGTLRELGQKYSDRLTVVHWLESVQGLPTATALAGLAAP
H37Rv_KshB PIMSICKSALAEGGGQVTLLYANRDDRSVIFGDALRELAAKYPDRLTVLHWLESLQGLPSASALAKLVAP
210 220 230 240 250 260 270
HGMS2_KshB122 FAAYEAFICGPGPFMSAAEAACKTVD..ARHIHIEVFKSLDSDPFAQVVIDVDEDDDRGPAQAIVELDGT
B-3805_KshB FAAYEAFICGPGPFMSAAEAACKTVD..ARHIHIEVFKSLDSDPFAQVVIDVDEDDDRGPAQAIVELDGT
KMS_KshB YTGHDAYICGPGPFMAAAEEALTSAGTAADKIHIEVFKSLESDPFAAVVIEED.DSDEGPATAVVTLDGE
MCS_KshB YTGHDAYICGPGPFMAAAEEALTSAGTAADKIHIEVFKSLESDPFAAVVIEED.DSDEGPATAVVTLDGE
H37Rv_KshB YTDRPVFICGPGPFMQAARDALAALKVPAQQVHIEVFKSLESDPFAAVKVDDS..GDEAPATAVVELDGQ
280 290 300 310 320 330 340
HGMS2_KshB122 THEIEWPRKAKLLDVLLNKGLDAPFSCREGHCGACAVLMRKGDVEMEINDVLEPSDLDEGLILACQALPT
B-3805_KshB THEIEWPRKAKLLDVLLNKGLDAPFSCREGHCGACAVLMRKGDVEMEINDVLEPSDLDEGLILACQALPT
KMS_KshB THEIRWPRNAKLLDVLLDKGLDAPFSCREGHCGACAVLKKSGEVEMAVNDVLEQSDLDEGLILGCQAMPR
MCS_KshB THEIRWPRNAKLLDVLLDKGLDAPFSCREGHCGACAVLKKSGEVEMAVNDVLEQSDLDEGLILGCQAMPR
H37Rv_KshB THTVSWPRTAKLLDVLLAAGLDAPFSCREGHCGACACTLRAGKVNMGVNDVLEQQDLDEGLILACQSRPE
350
HGMS2_KshB122 SDSVEVTYDE
B-3805_KshB SDSVEVTYDE
KMS_KshB SDSVEVTYDE
MCS_KshB SDSVEVTYDE
H37Rv_KshB SDSVEVTYDE

Figure S10. Amino acid Alignment of KshB122 from HGMS2 with other mycobacterial KshB enzymes. B-3805, *Mycobacterium* sp. NRRL B-3805; KMS, *Mycobacterium* sp. KMS; H37Rv, *Mycobacterium* sp. H37Rv and MCS, *Mycobacterium* sp. MCS. Filled and opened boxes indicated identical and similar residues, respectively.

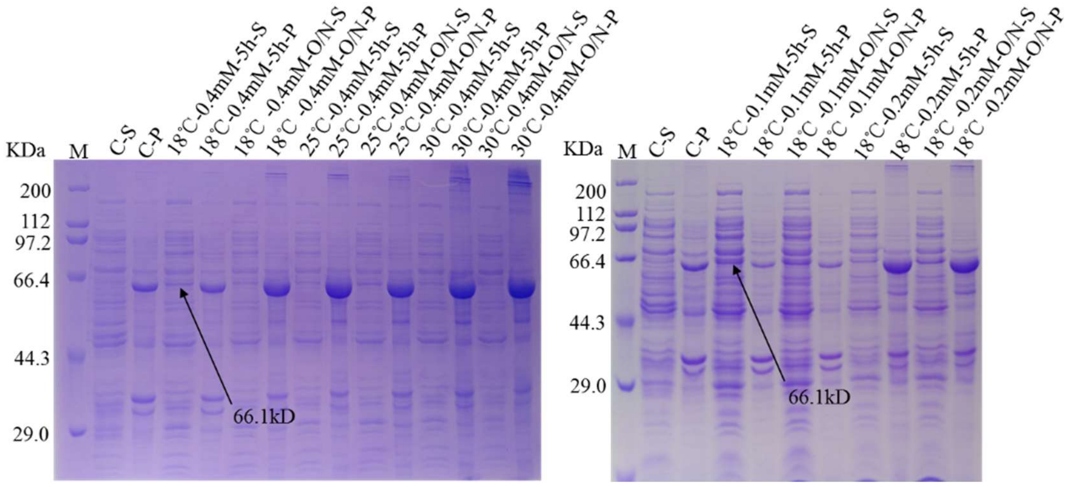

Figure S11. SDS-PAGE of small batch expression of His-tagged ChoM1 with different IPTG concentration at different induction temperature, indicating slight soluble protein. M, protein marker; C, control; S and P denote the supernatant and pellet of cell lysates, respectively. Arrows indicate expected proteins.

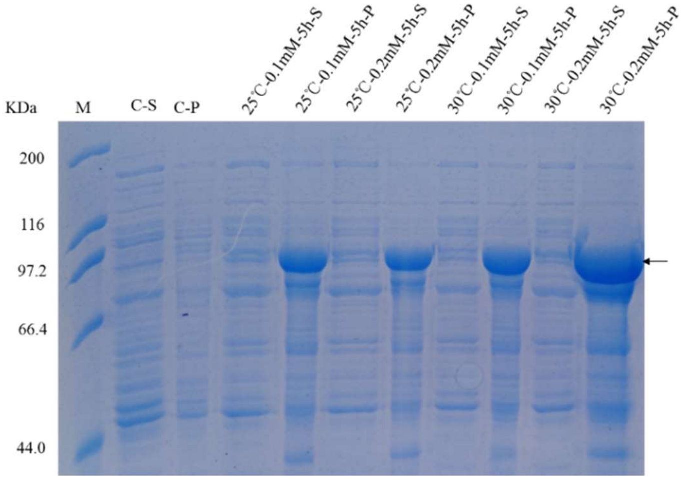

Figure S12. SDS-PAGE of small batch expression of GST-ChoM1 with different IPTG concentration at different induction temperature for 5 hrs, indicating no soluble protein. M, protein marker; C, control with no induction. S and P indicates respectively supernatant and pellet of the cell lysates. The arrow indicates expected protein.

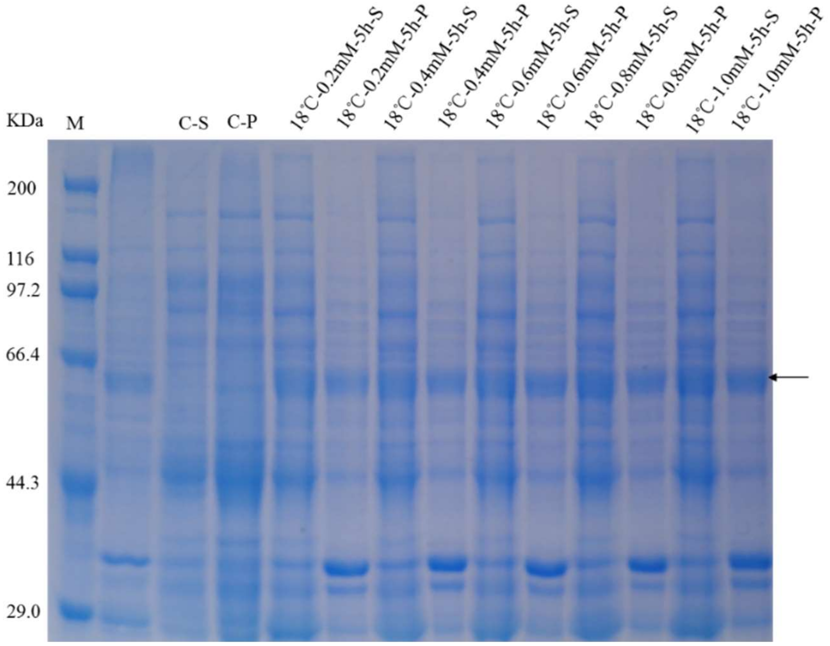

Figure S13. SDS-PAGE of small batch expression of GST-Hsd with different IPTG concentration at different induction temperature for 5 hrs, indicating no protein expression. M, protein marker; C, control with no induction. S and P indicates respectively supernatant and pellet of the cell lysates. The arrow indicates expected protein.

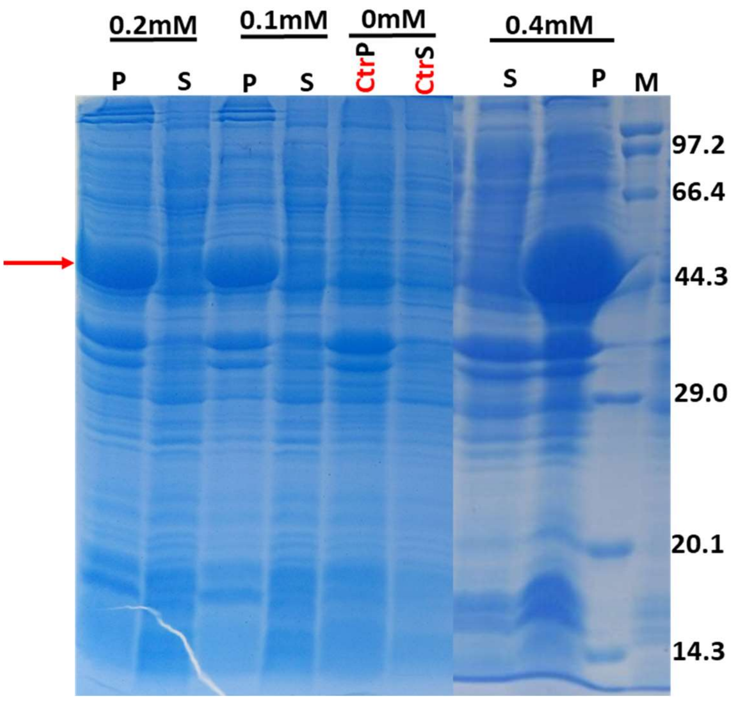

Figure S14. SDS-PAGE of small batch induced expression of His6-tagged Mon197, indicating no soluble protein. M, protein marker; S-beads, pulldown of supernatant; Ctro, control with no induction. S and P indicates respectively supernatant and pellet of the cell lysates. The arrow indicates expected protein.

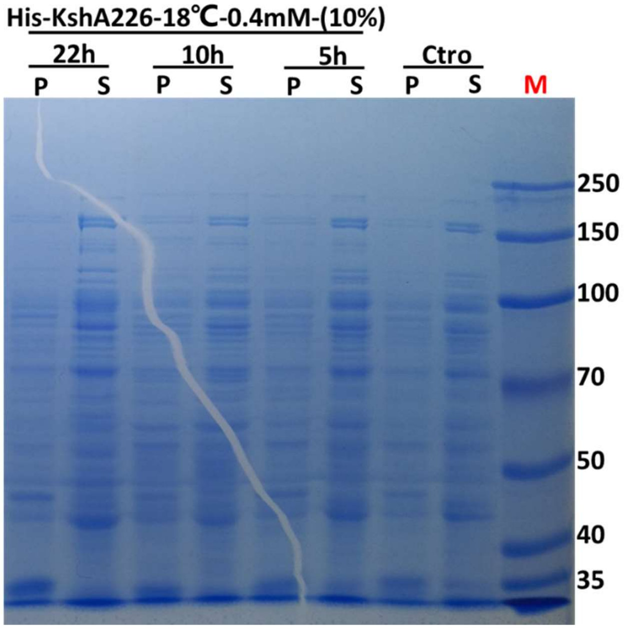

Figure S15. SDS-PAGE of small batch induced expression of His6-tagged KshA226, indicating no protein expression. M, protein marker; Ctro, control with no induction. S and P indicates respectively supernatant and pellet of the cell lysates.

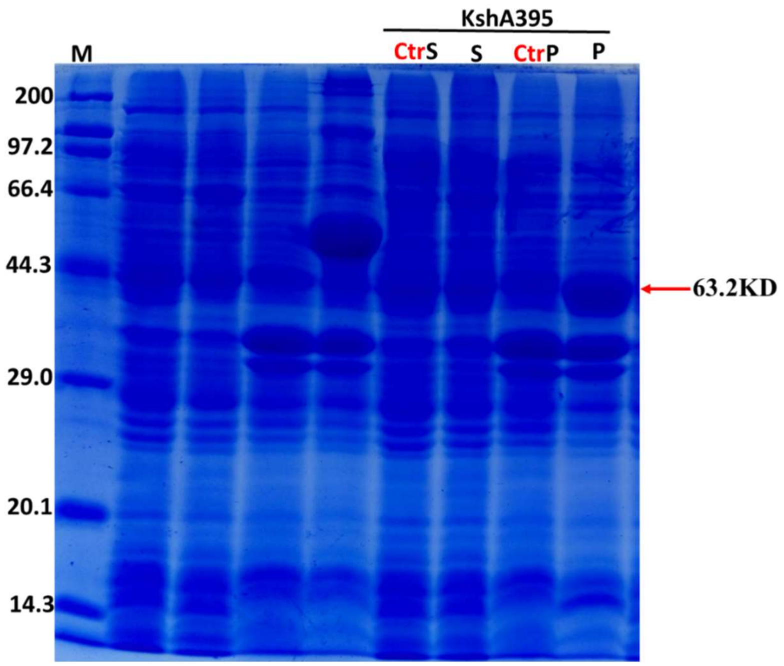

Figure S16. SDS-PAGE of small batch induced expression of His6-tagged KshA395, indicating no soluble protein. M, protein marker; Ctro, control with no induction. S and P indicates respectively supernatant and pellet of the cell lysates. The arrow indicates expected protein.

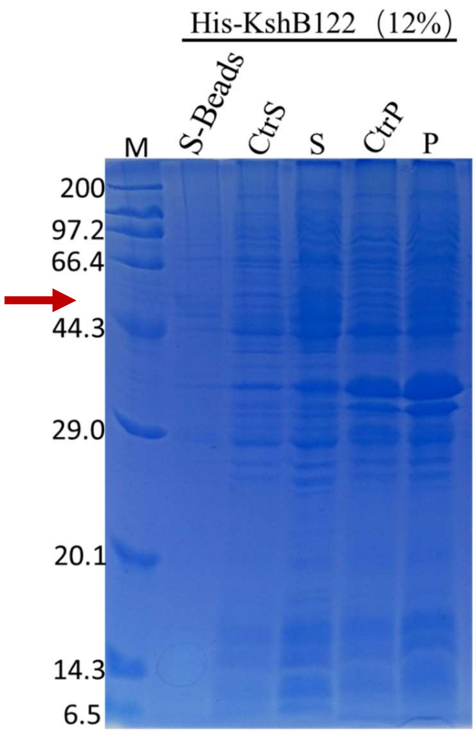

Figure S17. SDS-PAGE analysis of small batch induced expression of His6-tagged Ksh122 after pulldown with Ni-NTA beads, indicating no protein expression. M, protein marker; S-beads, pulldown of supernatant; Ctro, control with no induction. S and P indicates respectively supernatant and pellet of the cell lysates. The arrow indicates expected protein.

Reference

[1] Kanehisa M, Goto S, Kawashima S, Okuno Y, Hattori M (2004). The KEGG resource for deciphering the genome. Nucleic Acids Res 32 (Database issue): D277–80.

[2] Kanehisa M (1997). A database for post-genome analysis. Trends Genet 13 (9): 375–6.

[3] Kanehisa M, Goto S, Hattori M, Aoki-Kinoshita KF, Itoh M, Kawashima S, et al. (2006). From genomics to chemical genomics: new developments in KEGG. Nucleic Acids Res 34 (Database issue): D354–7.

[4] Tatusov RL, Koonin EV, Lipman DJ (1997). A genomic perspective on protein families. Science. Oct 24;278(5338):631-7.

[5] Tatusov RL, Fedorova ND et al. (2003). The COG database: an updated version includes eukaryotes. BMC Bioinformatics. Sep 11; 4:41.

[6] Magrane, M. and UniProt Consortium (2011) UniProt Knowledgebase: a hub of integrated protein data. Database (Oxford), bar009.

[7] Bard J, Winter R (2000). Gene Ontology： tool for the unification of biology. Nat Genet. 25:25-29.

[8] Vargas WA, Martín JM et al.(2012) Plant defense mechanisms are activated during biotrophic and necrotrophic development of Colletotricum graminicola in maize. Plant Physiol. 2012 Mar;158(3):1342-58.

[9] Liu B, Pop M. ARDB-Antibiotic Resistance Genes Database. Nucleic Acids Res. 2009 Jan;37(Database issue): D443-7.

[10] Chen LH, Xiong ZH, Sun LL, Yang J and Jin Q (2012). VFDB 2012 update: toward the genetic diversity and molecular evolution of bacterial virulence factors. Nucleic Acids Res. 40(Database issue): D641-D645.

[11] Cantarel BL, Coutinho PM, Rancurel C, Bernard T, Lombard V, Henrissat B (2009). The Carbohydrate-Active EnZymes database (CAZy): an expert resource for Glycogenomics. Nucleic Acids Res 37: D233-238.
